# Analysis of 30 chromosome-level *Drosophila* genome assemblies reveals dynamic evolution of centromeric satellite repeats

**DOI:** 10.1186/s13059-025-03527-4

**Published:** 2025-03-18

**Authors:** Daniel Gebert, Amir D. Hay, Jennifer P. Hoang, Adam E. Gibbon, Ian R. Henderson, Felipe Karam Teixeira

**Affiliations:** 1 Department of Genetics, University of Cambridge, Downing Street, Cambridge, CB2 3EH UK; 2 Department of Physiology, Development, and Neuroscience, University of Cambridge, Downing Street, Cambridge, CB2 3DY UK; 3 Department of Plant Sciences, University of Cambridge, Downing Street, Cambridge, CB2 3EA UK; 4 Present Address: Department of Molecular and Cell Biology, University of California, Berkeley, CA USA

## Abstract

**Background:**

The *Drosophila* genus is ideal for studying genome evolution due to its relatively simple chromosome structure and small genome size, with rearrangements mainly restricted to within chromosome arms, such as Muller elements. However, work on the rapidly evolving repetitive genomic regions, composed of transposons and tandem repeats, have been hampered by the lack of genus-wide chromosome-level assemblies.

**Results:**

Integrating long-read genomic sequencing and chromosome capture technology, here we produce and annotate 30 chromosome-level genome assemblies within the *Drosophila* genus. Based on this dataset, we reveal the evolutionary dynamics of genome rearrangements across the *Drosophila* phylogeny, including the identification of genomic regions that show comparatively high structural stability throughout evolution. Moreover, within the *ananassae* subgroup, we uncover the emergence of new chromosome conformations and the rapid expansion of novel satellite DNA sequence families, which form large and continuous pericentromeric domains with higher-order repeat structures that are reminiscent of those observed in the human and *Arabidopsis* genomes.

**Conclusions:**

These chromosome-level genome assemblies present a valuable resource for future research, the power of which is demonstrated by our analysis of genome rearrangements and chromosome evolution. In addition, based on our findings, we propose the *ananassae* subgroup as an ideal model system for studying the evolution of centromere structure.

**Supplementary Information:**

The online version contains supplementary material available at 10.1186/s13059-025-03527-4.

## Background

Shortly after the introduction of the fruit fly (*Drosophila melanogaster*) as a model organism for genetic research, the cornerstone for genomic and chromosome studies was laid by the creation of the first genetic map of the X chromosome [1]. The subsequent description of chromosomal structural rearrangements [2], and the following studies on their implications for genetic inheritance and evolution [3–5], firmly established the fruit fly as the prime model for the study of chromosome structure evolution.

The *Drosophila* genus is particularly suited for research on genome evolution due to the small number of relatively short chromosomes (~ 1.3–61 Mb in *D. melanogaster*) [6], which display organization into gene-rich euchromatic arms and gene-poor heterochromatic pericentromeric regions that are populated by diverse families of transposable elements (TEs) [7]. Pioneering comparative cytogenetic studies throughout the *Drosophila* genus revealed that genomic rearrangements mostly occur within chromosome arms, with inter-chromosomal rearrangements being rarely observed, an insight that was further expanded by the analyses of the first genome drafts of 12 *Drosophila* species [5, 8, 9]. These chromosome arms, representing genomic units with overall relatively consistent genetic material, have been dubbed Muller elements [5, 10, 11]. The six Muller elements are classically named from A to F, and respectively correspond to chromosome arms X, 2L, 2R, 3L, 3R and 4 in *D. melanogaster*. Importantly, the near lack of translocations between Muller elements presents an exceptional opportunity to follow genomic annotations and rearrangements through evolution within a genus. Furthermore, this feature of genome evolution facilitates the analysis of chromosome structure: for instance, while elements B (2L) and C (2R), as well as D (3L) and E (3R), are fused in *D. melanogaster* to form two metacentric chromosomes, other lineages have evolved completely different chromosome organizations with different arrangements of the six Muller elements [12–14].

While the gene content of Muller elements is relatively stable, the repetitive parts of the genome evolve quickly and in more unrestrained ways [8]. Two major types of repetitive sequences, namely TEs and satellite DNA, are primarily located in the *Drosophila* pericentromeric heterochromatin regions [6]. However, despite being enriched within heterochromatic genomic territories, TEs are mobile elements that are otherwise not restricted to a given Muller element [15]. Moreover, pioneering work in *Drosophila* uncovered that some TE families can break the species barrier, horizontally transferring to other related species and spreading quickly through wild populations [16–18]. Similarly dynamic, the composition and organization of tandem-repetitive satellite DNA are complex and highly variable, frequently leading to species-specific satellite DNA families [19]. For instance, while the centromere sequences on all human chromosomes are mostly composed of highly repetitive 171-base pair (bp) alpha satellites organized into mega base pair (Mb)-long arrays of higher-order repeats (HORs) [20], *D. melanogaster* centromeres consist of retrotransposon-rich DNA blocks flanked by arrays of short simple repeats (5–12 bp) [21]. Also, *D*. *melanogaster* centromeres, as defined by the domain enriched for the deposition of centromere-specific histone H3 variant, are much shorter than in humans (101–171 kb), with sequence and TE composition substantially differing between chromosomes [21]. While little is known about the centromeres of other species within the *Drosophila* genus, estimates of the share of genomic satellite DNA suggest a wide disparity between species [22]. Beyond that, satellite arrays with much larger monomer lengths (90–500 bp) than those observed in *D. melanogaster* have been identified in closely related species [23].

TE and satellite dynamics are central to understanding genome evolution, but our capacity to study these processes has been hampered by the fact that only a few species across the *Drosophila* genus have a chromosome-level genome assembly [6, 13, 14, 24, 25], and these are rarely coupled with detailed analyses focusing on the repetitive parts of the genome [26]. Indeed, most existing assemblies for the other *Drosophila* species are either almost exclusively composed of euchromatic sequences and lack the repetitive content of the genome [9] or are comprised of unordered and fragmented contigs generated by long-read sequencing [27–29]. While the recent advances in long-read sequencing technology, such as Oxford Nanopore Technology (ONT), have enabled the assembly of much more complete genomes, most of the genome assemblies still do not reach chromosome-level quality. One solution to circumvent the remaining assembly challenges is to combine long read and chromosome capture (HiC-seq) data to exploit the underlying information on chromatin interactions to scaffold contigs into chromosomes [30–32].

Here, we have used HiC sequencing in 30 *Drosophila* species that were part of recent ONT-based sequencing efforts to produce high-quality chromosome-level genome assemblies across the *Drosophila* genus. Thanks to these highly contiguous assemblies and refined genome annotations of genes, TEs, and satellite DNA, we were able to describe the evolution and dynamics of structural rearrangements across the phylogeny. Based on this, we identified chromosomal regions that stand out as unusually stable over evolutionary time, thereby uncovering gene clusters that provide new models for studying gene function and regulation. Additionally, at the chromosome level, our analyses revealed how different chromosomal evolutionary paths can lead to the de novo appearance of metacentric Muller elements from otherwise acrocentric/telocentric structures. Finally, a detailed analysis of satellite DNA sequences allowed us to uncover an extraordinary evolutionary burst of complex satellite repeats in the *ananassae* subgroup. This expansion is defined by the emergence of large stretches of highly structured satellite DNA repeat arrays within the expected peri/centromeric regions, together with an expansion of distinct DNA satellite families within the euchromatic arms of the Muller elements. The remarkable emergence and radiation of peri/centromeric complex satellite DNA, the distribution and organization of which are more reminiscent of the centromeric structures in humans and *Arabidopsis* compared to other *Drosophila* species, establishes the *ananassae* subgroup as a new model for studying the evolution of satellite DNA and centromeres in metazoans.

## Results

### Genome scaffolding and annotation

**Fig. 1:**
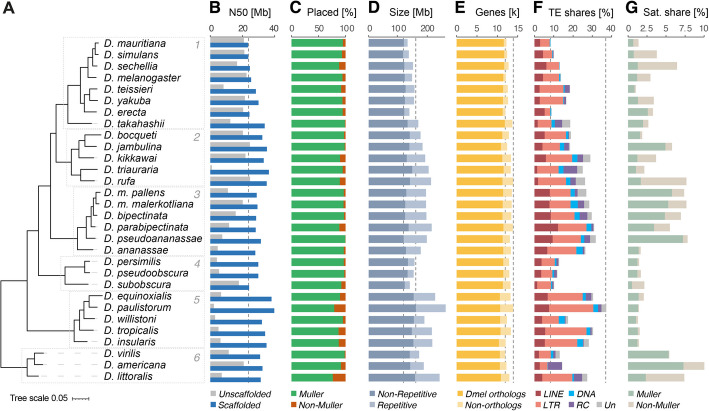
Overview of assembly quality, Muller placement, size, and annotation of genes, TEs, and satellites.** A** Phylogenetic tree produced by OrthoFinder based on the consensus of all gene trees. Species subgroups are numbered 1–6. Tree scale shows (0.05) rate of substitutions per amino acid site. **B** N50 values of unscaffolded assemblies published by Kim et al. [28] (gray) and assemblies scaffolded in this study (blue) in Mb (million base pairs). The dashed line indicates the minimal N50 value after scaffolding (*D. mauritiana*) among all 30 species. **C** Percentage of genomic sequence placed within Muller element scaffolds (green) or unplaced scaffolds (orange). **D** Size of scaffolded genome assemblies in Mb. Non-repetitive DNA shown in dark shade, repetitive DNA shown in light blue. **E** Number of thousands of genes with (dark) and without (light) identified *D. melanogaster* orthologs. The dashed lines indicate the minimal and maximal non-repetitive genome sizes. **F** Share of annotated transposable elements (TE) classes as percentage of genomic sequence. **G** Share of satellite DNA as percentage of genomic sequence that are located within Muller element scaffolds (green) or unplaced scaffolds (beige)

In order to improve the contiguity of genome assemblies that were generated through Oxford Nanopore Technology (ONT) sequencing [27, 28], and arrange contigs into chromosomes, we have leveraged publicly available data [13, 14, 24, 25, 33–35], and generated HiC data from female flies for a total of 30 *Drosophila* species spanning ~ 40–62 million years of evolution [36–38]. For this analysis, we have divided these 30 species into six subgroups according to their phylogeny, with subtrees of more closely related species (Fig. 1A). Using the HiC chromatin contact information within a streamlined computational pipeline (see “Methods”; [39]), followed by manual curation, we were able to scaffold the contigs into highly contiguous, chromosome-level genome assemblies.

To assess the quality of the new assemblies, we compared those of D. melanogaster and its closest relatives to other assemblies that have been recently generated using long-read sequencing methods [21, 23], as well as to the *D. melanogaster* reference genome (*dm6*). The new *D. melanogaster* assembly showed higher contiguity and completeness, in particular in the repeat-rich regions of each chromosome (i.e., pericentromeric heterochromatin), when compared to the assembly recently generated using PacBio long-read technology without any further scaffolding step (Additional file 1: Fig. S1A-C) [21]. Whole genome alignments also showed that the new assembly is very similar to the *D. melanogaster* reference genome *dm6* in both sequence and contiguity (Additional file 1: Fig. S1C, S5C) and, yet, shows higher contiguity in the repeat-rich regions of the genome. A similar trend was observed for the other species for which we could perform direct comparisons. Indeed, the new genome assemblies for *D. sechellia*, *D. simulans*, and *D. mauritiana* displayed similar contiguity compared to the corresponding assemblies that were recently generated using PacBio long-read technology and scaffolded using *D. melanogaster* as a reference (Additional file 1: Fig. S1D,E) [23]. However, the use of *D. melanogaster* genome for scaffolding the long-read sequencing data from heterologous (yet closely-related) species can lead to assembly inconsistencies, in particular in the repeat-rich regions of the genome (Additional file 1: Fig. S1E).

As revealed by comparing N50 values, the use of HiC data led to a substantial improvement of contiguity, with some species showing a quality increment of more than 25-fold. While the N50 of the unscaffolded assemblies ranged from 0.9 to 24.7 Mb, with a median of 12.0 Mb, the finished assemblies had an N50 of at least 23.5 Mb and up to 39.8 Mb, with a median of 30.4 Mb (Fig. 1B). The increase in the scaffolded N50 was even more drastic when considering “heterochromatic” sequences alone (see methods), while “euchromatic” N50 values were slightly improved in some species and quite substantially in others (Additional file 1: Fig. S1F). Importantly, we did not observe any correlation between the N50 values of the unscaffolded assemblies and those of the finished assemblies, and our results indicate that the quality of the initial assemblies did not influence the capacity of scaffolding the contigs into highly contiguous assemblies when using HiC data.

As chromosome naming conventions vary substantially between species, even for homologous chromosome arms, we have organized each assembled genome according to the Muller element nomenclature [10, 11] by consistently allocating the same name (elements A–F) to homologous chromosome arms across species. This was achieved through whole genome alignment against the *D. melanogaster* reference genome (dm6), in which chromosome arms X, 2L, 2R, 3L, 3R, and 4 correspond to Muller elements A, B, C, D, E, and F, respectively. To orientate the chromosome arms, we have used the asymmetry of the transposon-rich ends of Muller elements as an anchor to identify the presumed pericentromeric region. The proportion of remaining scaffolds or contigs that could not be placed into any Muller element was consistently low, although it varied between species (Fig. 1C). Indeed, the share of genomic sequence allocated to Muller elements ranged from 79.1 to 99.5%, with a median value of 94%. Importantly, we noticed that the proportion of unassembled contigs in a given species correlated with the sex of the adult flies sampled for Nanopore sequencing [27, 28], with samples containing males showing a slightly higher share of the genome that was not placed into any Muller element (Additional file 1: Fig. S1G; Additional file 2: Table S1). Together with the fact that most of the HiC data was generated using female flies (Additional file 2: Table S1), these results suggest that the remaining unscaffolded contigs are likely to be enriched for sequences derived from the male-specific Y-chromosome.

Total genome size varies between 130 and 257 Mb (mean 179 Mb; standard deviation 32.2 Mb) and correlates well with estimated genome sizes, regardless of the estimation method used (Additional file 1: Fig. S1H). This range in size reduces when considering only non-repetitive sequences (119 to 172 Mb; mean 139 Mb; standard deviation 13.5 Mb), indicating a strong influence of repeat content on variation in genome size (Fig. 1D). Consistently, the gene content is more stable than genome size across species. Here, genes were identified de novo with MAKER [40] by coupling predictive tools, homology-dependent comparisons, and RNA-seq data that we generated from dissected ovaries for each of the 30 *Drosophila* species. The number of identified genes ranged from 11,893 to 13,880 per species, with a mean of 12,914 genes (Fig. 1E). On average, for ~ 86.9% of the genes identified in a given species, a homolog could be identified in *D. melanogaster*, with more distantly related species presenting less shared gene content.

For the annotation of genomic repeat content, we used specialized methods for TEs and satellite DNA. Transposons were identified using HiTE [41], which applies dynamic boundary adjustment to detect full-length genomic copies. Satellite DNA, on the other hand, was annotated using the Tandem Repeat Annotation and Structural Hierarchy (TRASH) [42], which identifies regions containing tandem repeats and their consensus monomer sequences. These analyses showed that not only the total share of TE sequence varied substantially between genomes, but also the representation of TE classes (Fig. 1F). As previously described [43, 44], we found a positive correlation between genome size and transposon content in the *Drosophila* genus. Differences among subgroups of species became apparent when considering the phylogeny of the 30 *Drosophila* species. For example, the members of subgroup 3 (*ananassae*) have consistently high proportions of TEs (26.6–31.8%) with a similar class composition, while the genomes of species in subgroup 4 (*obscura/pseudoobscura*) are relatively depleted of TEs (10.2–12.8%; Fig. 1F). Interestingly, a similar pattern can be observed for the genomic share contributed by tandem repeats, i.e., satellite DNA, although not as strongly correlating with genome size as TE content (Fig. 1G). Again, subgroup 3 (*ananassae*; median of 7.2%), together with subgroup 6 (*virilis;* median of 7.4%), stands out with a high amount of tandem-repetitive DNA content, in addition to TEs.

### Chromosomal genome organization

**Fig. 2:**
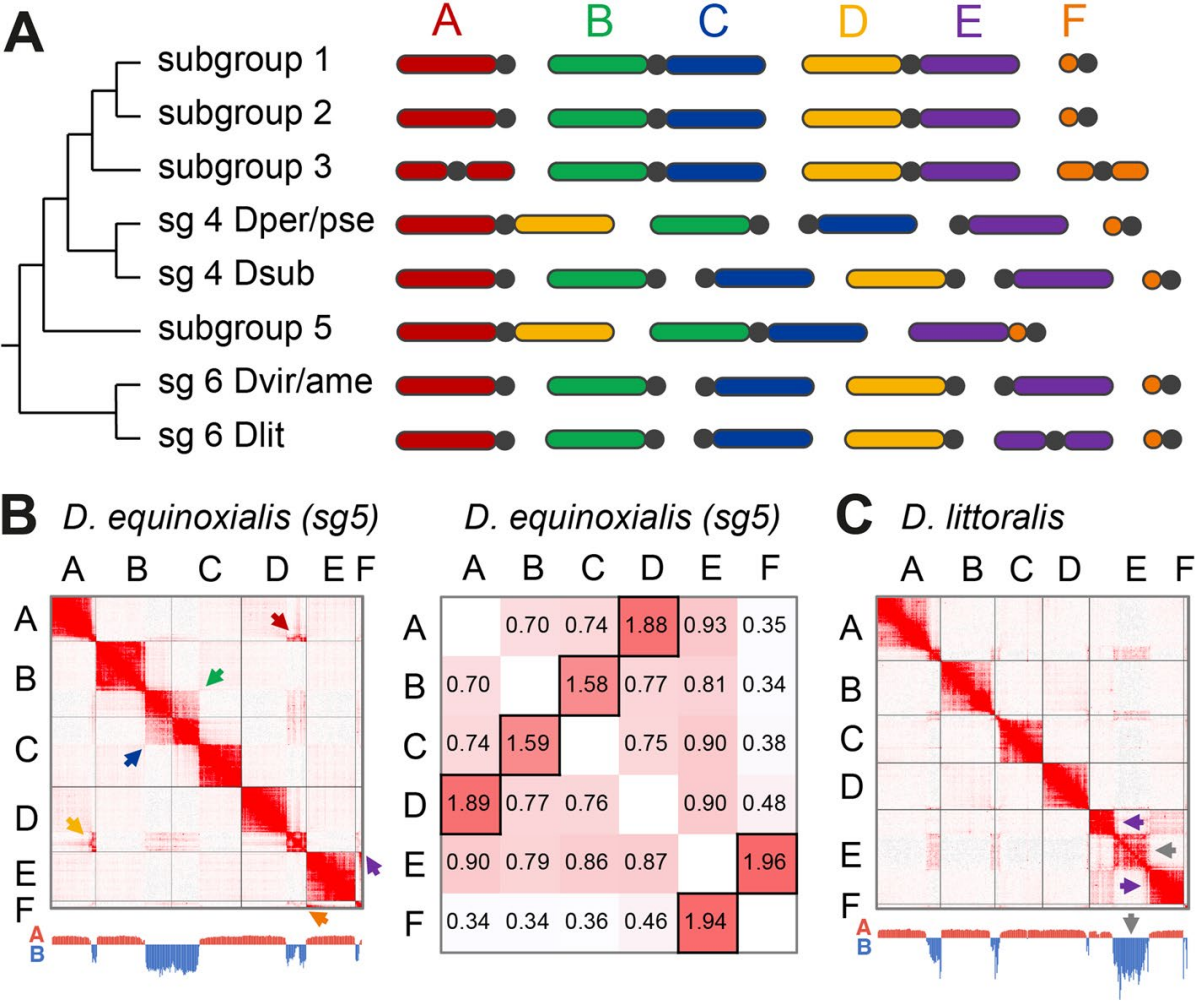
The chromosomal organization of Muller elements in different species subgroups.** A** Schematic of Muller element chromosomal organizations. Chromosome bodies are colored according to Muller element and centromeres are represented by black dots. **B** Left panel: HiC contact map of Muller elements for *D. equinoxialis* (subgroup 5). Contacts between different Muller elements are highlighted by arrows: **B** and **C** contacts in green and blue arrows; **A** and **D** contacts in red and yellow arrows; **E** and **F** contacts in purple and orange arrows. Distribution of A/B compartment along the genome is shown below the HiC maps (whole genome PCA eigenvectors; for Muller element-specific PCA, see Additional file 1: Fig. S2). Right panel: Normalized contact intensity values between different Muller elements. Individual squares for each pairwise comparison are colored on a white-to-red scale representing low-to-high contact values. Squares containing the highest values for each pair of Muller elements are highlighted by black borders. **C** HiC contact map of Muller elements for *D. littoralis* (subgroup 6). Purple arrows highlight A compartment of Muller E; gray arrows highlight B-compartment

In addition to allowing contig scaffolding, the HiC data provides information for the physical contacts between Muller elements and how these elements may be organized into chromosomes. For each species, we have quantified inter-Muller element contacts (per kb) to reconstruct chromosomal genome organization (Fig. 2). Out of the 30 species, eight genome configurations can be distinguished, which mostly follow the divisions according to subgroups (Fig. 2A). Subgroups 1–3 generally have the same chromosomal organization as *D. melanogaster*, with B and C elements organized in a metacentric chromosome structure, and D and E elements forming another metacentric chromosome. However, and in contrast to subgroups 1 (*melanogaster/takahashii*) and 2 (*montium*), in which chromosomes A and F are acrocentric, all members of subgroup 3 (*ananassae*) contain metacentric versions of each of these chromosomes. In addition, in subgroup 3, both chromosomes A and F are also greatly enlarged, which agrees with earlier cytogenetic analyses [12]. Analysis of chromatin compartments, using principal component analysis (PCA), shows that extensive amounts of B-compartment chromatin, which is most likely heterochromatic and repeat-rich [45], are located in the center of these chromosomes. These structures contrast to those observed for subgroups 1 and 2, in which B-compartment chromatin is found at the tips of both chromosomes A and F.

Hi-C analysis also confirmed previous cytogenetic analysis revealing that A and D elements are found in a fused chromosomal conformation in both subgroups 4 (with the exception of *D. subobscura*) and 5 (*willistoni*), forming an A-D metacentric chromosome. Such a fusion is particularly unusual, since sex chromosomes, such as Muller element A, and autosomes have distinct evolutionary trajectories, recombination rates, and patterns of gene expression [46–48]. Interestingly, following the principle of parsimony, it seems most likely that the A-D fusions in subgroups 4 and 5 occurred independently, instead of a single fusion event followed by at least two separations in *D. subobscura* and the *melanogaster* group. As this scenario is at odds with the presumed reduced likelihood of such an event to occur in the first place, it indicates a role for selection. Finally, in subgroup 5, we also observed an attachment of F to E element (Fig. 2B, Additional file 1: Fig. S2A), which creates an acrocentric E–F chromosome.

Notably, we observed that all Muller elements are separate from each other in *D. subobscura* (subgroup 4), and all species in subgroup 6 (*virilis*), forming their own chromosomes. These are all acrocentric with the single exception of *D. littoralis*, which possesses a metacentric E element (Fig. 2C, Additional file 1: Fig. S2B). As we observed for the A element in subgroup 3 (*ananassae*), the newly metacentric E element in *D. littoralis* contains a large (~ 20 Mb) stretch of B-compartment chromatin in the middle of the chromosome, which is expected to represent the pericentromeric region and the region encompassing the centromere. Finally, we observed that in *D. littoralis*, elements B and D show elevated contacts per kb values, although these are not distinct enough compared to background levels to reliably indicate a physical connection in vivo (Additional file 1: Fig. S2B).

### Genomic rearrangements and the evolution of chromosome structure

**Fig. 3:**
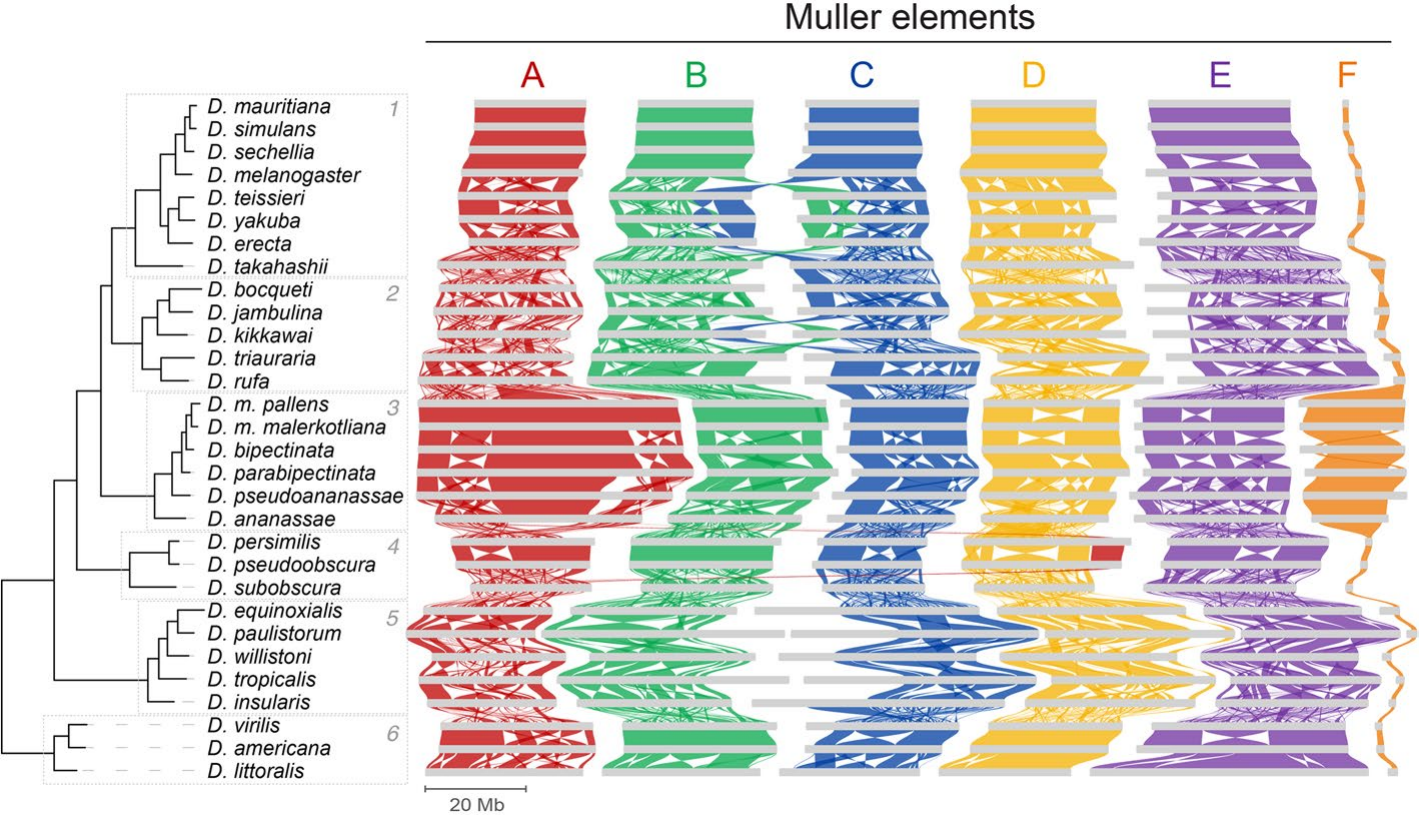
Overview of genomic rearrangements across *Drosophila *species. Ribbons between Muller elements of different species represent syntenic blocks based on gene synteny. The F elements of *D. pseudoananassae*, *D. ananassae*, *D. persimilis*, and *D. pseudoobscura* were reversed for visual clarity

Based on the de novo gene annotations and using the software package GENESPACE [49], we have reconstructed a detailed evolutionary history of the genome rearrangements occurring across the phylogenetic tree (Fig. 3). As expected, exchanges of genetic material between Muller elements were very rare and restricted to a few examples. First, we observed a previously described pericentric inversion between the physically linked B and C elements [12, 50] in the lineage leading to *D. erecta*, *D. yakuba*, and *D. teissierei* (subgroup 1). Our analysis reveals that the initial inversion was followed by subsequent rearrangements of B element genes deeper into the C element of these species, and vice versa. Coincidentally, another pericentromeric inversion between B and C elements happened independently in *D. kikkawai* (subgroup 2). Except for these B-C exchanges, the only other example of an exchange between Muller elements was observed in *D. persimilis* and *D. pseudoobscura* (subgroup 4). Following the fusion of elements A and D to form a metacentric chromosome in the ancestor of these two species, a large stretch of the A element DNA was transferred to the pericentromeric end of the D element. This may have resulted from the relocation of the centromere after fusion, rather than a pericentric inversion, since no converse transfer from D to A could be observed (Additional file 1: Fig. S3) [51]. Alternatively, a pericentric inversion with a breakpoint very close to the centromere could explain the observed structure as well. Importantly, our analyses confirm that, in all cases where large exchanges of genetic material were observed, these involved Muller elements that were already physically attached to form a single chromosome structure.

**Fig. 4:**
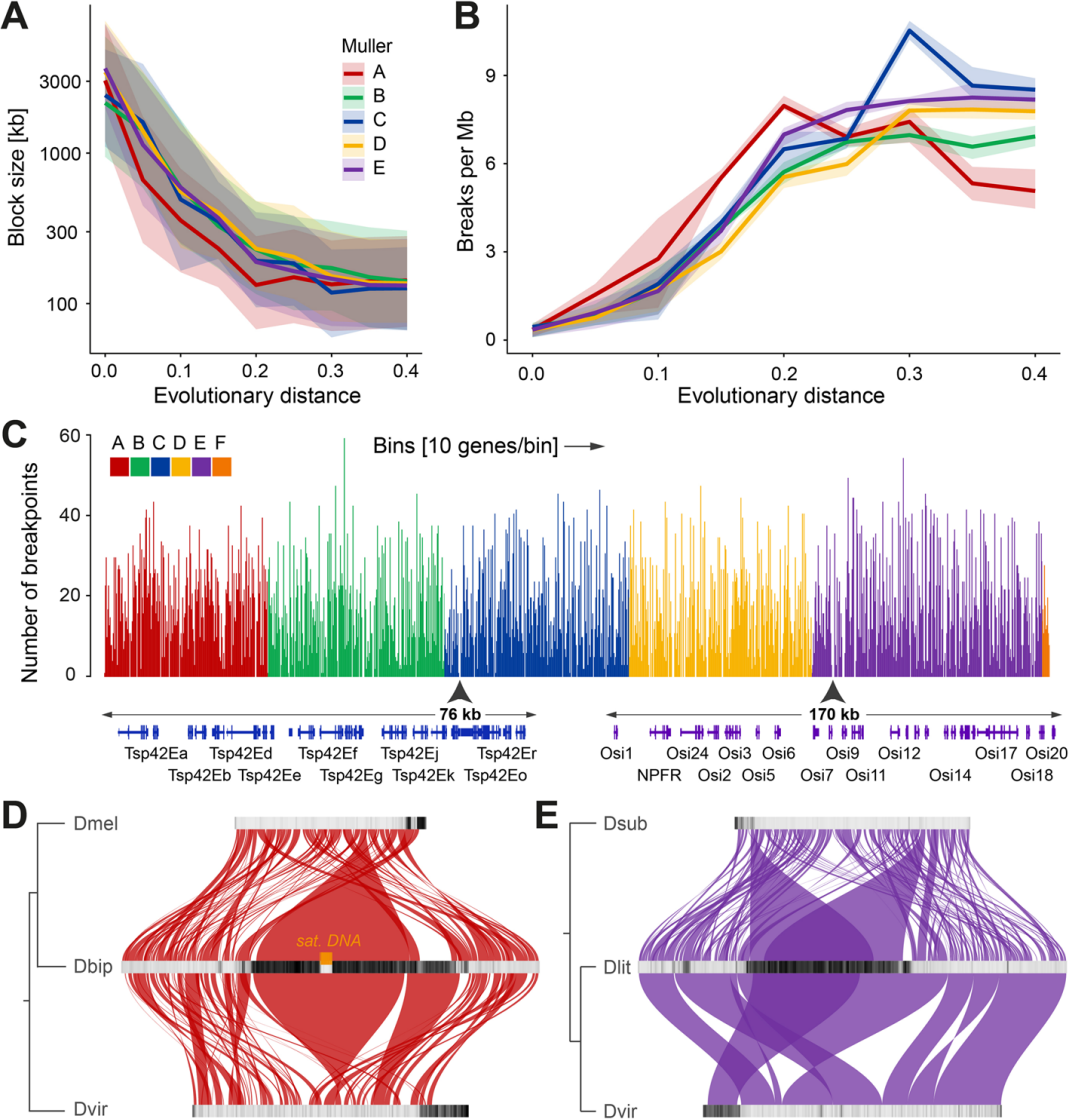
Evolutionary analysis of genomic rearrangements and syntenic blocks.** A** Syntenic block sizes in kilo base pairs (kb) relative to evolutionary distance in substitutions per amino acid site for each Muller element. Ribbons around lines represent interquartile range (IQR). **B** Breaks between synteny blocks per million base pairs (Mb) relative to evolutionary distance for each Muller element. Ribbons around lines represent IQR. **C** Number of synteny breakpoints within bins of 10 genes across the *D. melanogaster* genome when compared to the remaining 29 species. One thousand one hundred ninety eight gene bins are shown continuously in the order as they are located on Muller elements from **A** to **F**. Zoomed-in genomes browser views of gene cluster of *Tetraspanin 42E* and *Osiris* gene families are shown below, as representatives of prominent sites with multiple consecutive bins devoid of breakpoints. **D** Synteny between A elements of *D. bipectinata* compared to *D. melanogaster* and *D. virilis*. TE densities per 200-kb bins are shown with black (high TE density) to light gray (low TE density) scales. **E** Synteny between E elements of *D. littoralis* compared to *D. subobscura* and *D. virilis*

Based on our genomic rearrangement analysis, centered around GENESPACE output, we have estimated the distribution of syntenic block sizes, which have a median range of 149 to 171 kb between Muller elements. Relying on protein sequence divergence to define the evolutionary distance (amino acid substitutions per site) between all possible pairs of species, we observed that the average syntenic block size decreases rapidly during evolution, following an exponential decay curve (Fig. 4A). Further, to better visualize the rates of genomic rearrangement, we determined the number of breaks per Mb per Muller element as a function of evolutionary distance (Fig. 4B). After an initial exponential surge in the number of breaks per Mb per evolutionary distance, we observed a saturation point just below 10 breaks per Mb at greater evolutionary distances. Notably, this inflection point coincides with the maximum distance between species in a given subgroup, indicating that instead of being a feature of genome evolution, these results may instead indicate a methodological limit to the sensitivity of detecting rearrangements at increased evolutionary distances.

Having this baseline view of genomic rearrangement rates through evolution, we examined cases that show deviations from the background expectation. A clear example is presented by the *Hox* genes clusters ANT-C and BX-C that are found on the E element. Evolutionary tracing revealed that these clusters are not subject to internal rearrangements, despite the drastic changes observed when considering their chromosomal position (Additional file 1: Fig. S4A). Using a more systematic approach, we determined the number of synteny breakpoints across the *D. melanogaster* genome compared to the remaining 29 species (Fig. 4C). In order to reduce the bias that might affect gene-poor regions, we considered the number of breakpoints within bins of 10 genes instead of using genomic distance as measured in base pairs. The absence or small number of breakpoints per bin indicates groups of genes that are unlikely to be separated throughout evolution. Upon closer inspection, several larger genomic stretches devoid of synteny breakpoints could be identified. Remarkably, many of these were defined by gene family clusters, such as the *Osiris* cluster (*Osi1-20*) on the E element. The conservation of this gene cluster has been shown to not only be restricted to flies but to also apply to other insect orders [52]. Another example is provided by a cluster of *Tetraspanin* genes (*Tsp42Ea-r*) on the C element, of which little is known beyond the fact that these genes encode transmembrane proteins implicated in cell–cell interactions and signalling pathway regulation [53, 54]. While it is unclear what function these genes provide, their constrained synteny across the *Drosophila* genus suggests that these genes act in concert, with a strong selective pressure against rearrangements. Beyond these examples, our analyses revealed many other cases of deep synteny conservation (e.g., Ccp84Aa-g and alpha-Est1-10; Additional file 3: Table S2) [55, 56], which are likely to represent new models for understanding gene cluster function and regulation.

### Evolution of metacentric Muller elements

The newly metacentric A element (X chromosome) of subgroup 3 (*ananassae*) is an interesting study case in the context of the evolution of chromosome structures. The appearance of a new centromeric-like block in the middle of the chromosome created two arms, called chrXL and chrXR, each behaving as a novel Muller-like element [57]. Indeed, and similar to what is observed for the classical Muller elements, we have not observed any rearrangement involving chrXL and chrXR (Fig. 3). On the other hand, during the same time scale, numerous inversions and other rearrangements were observed within each A-compartment (euchromatin) of the newly formed X chromosome arms. These results indicate that the new chromosome arms behave similarly to other metacentric chromosome structures, revealing that the de novo appearance of the pericentromeric block was determinant for defining the dynamics of chromosome rearrangements.

To better characterize the process leading to the de novo appearance of internal centromeric blocks within otherwise acrocentric Muller elements, we focused on the analysis of the A element of subgroup 3. Comparison of the *D. bipectinata* (subgroup 3) X chromosome to the homologs of *D. melanogaster* and *D. virilis* (outgroups) revealed that the repeat-rich pericentromeric regions of both outgroup species show no homology to the vast pericentromeric region in the metacentric A element of *D. bipectinata* (Fig. 4D). Instead, this expanded region, which likely contains the new centromere, due to a large TE-depleted gap that is instead highly enriched in satellite DNA, shows homology to more central, euchromatic parts of the A elements in *D. melanogaster* and *D. virilis.* This result suggests that rather than the relocation of the acrocentric centromere to the middle of the chromosome via rearrangements, the current structure of the metacentric A element in subgroup 3 was defined by the appearance and expansion of an internally located, new centromeric region.

As mentioned before, another example of a large Muller element that has become metacentric is provided by the E element of *D. littoralis*. Here, we compared it to *D. virilis* and *D. subobscura* (outgroups), both of which also have an individualized, acrocentric E element (Fig. 4E). In this case, the metacentric region of the E element in *D. littoralis* shares homology with the pericentromeric segments located at the edges of the chromosomes in other species. This is especially clear when *D. littoralis* is compared to its close relative *D. virilis*, but it is also apparent when examining more distantly related species such as *D. subobscura*. These results suggest that, in this case, the euchromatic regions may have exchanged places with the heterochromatic segments via chromosome rearrangements, leading to the internalization of the centromere. However, it is important to note that other euchromatic parts of both *D. subobscura* and *D. virilis* show homology with the heterochromatin in *D. littoralis*, although these alternative synteny block pairs share a lower number of orthologs and therefore are less supported (Additional file 1: Fig. S4B). More broadly, our analyses reveal that different chromosomal evolutionary paths could lead to the de novo appearance of metacentric Muller elements.

### Genomic distribution of TEs and satellite DNA

**Fig. 5:**
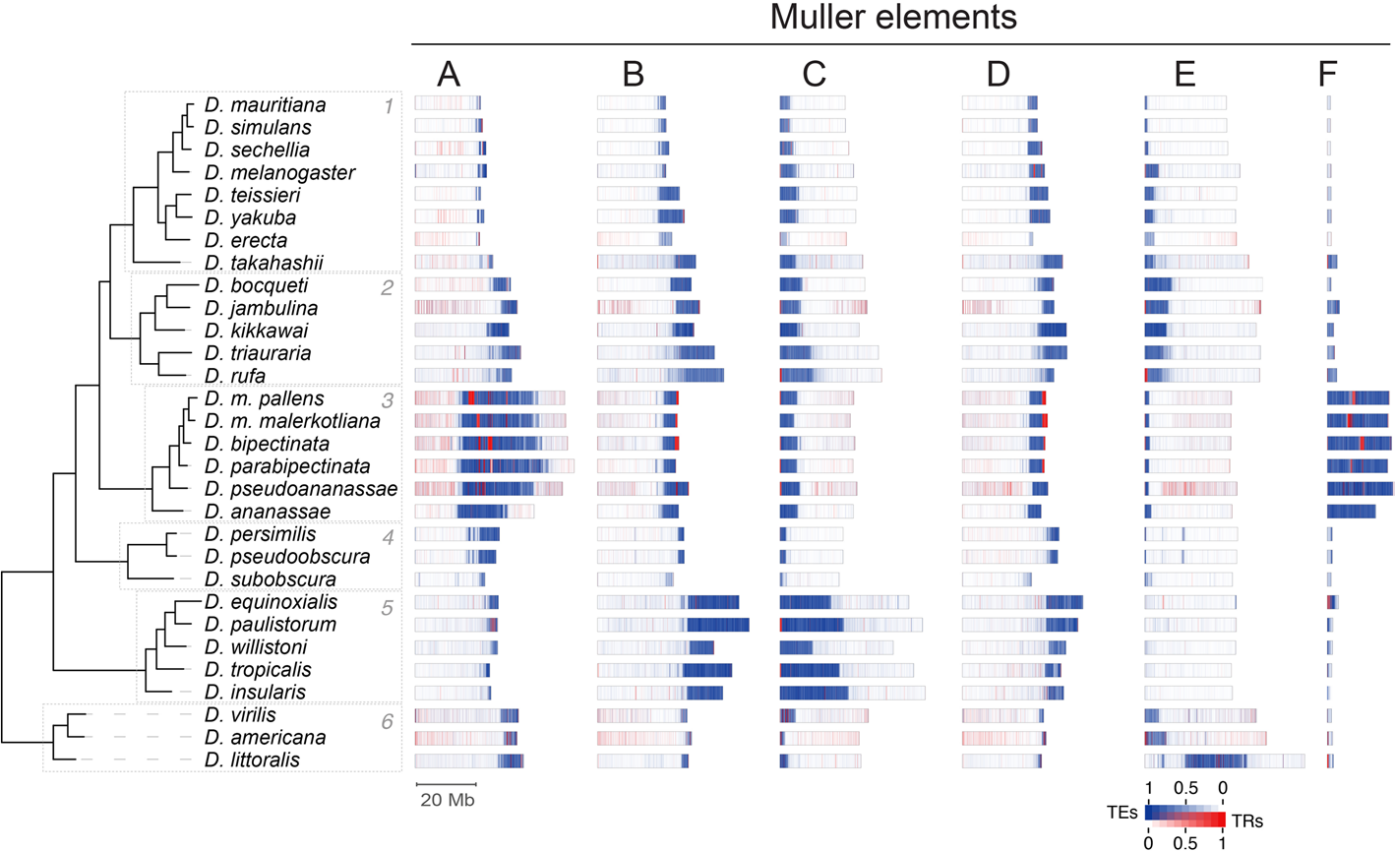
Distribution of TEs and satellite DNA across Muller elements. Density of TEs (blue) and satellite DNA (red) are shown in bins of 100 kb. Satellite DNA includes both simple and complex sequences

Throughout the *Drosophila* genus, we observed that the distribution of TEs across Muller elements is closely linked to chromosomal organization. Indeed, TEs are highly concentrated within the pericentromeric regions, i.e., close to the centromere, whereas TE frequency reduces precipitously at the transition from heterochromatin to euchromatin (Fig. 5). On the other hand, the frequency and distribution of satellite DNA repeats, as identified by the TRASH package [42], is much less pronounced, with only a few species showing clearly defined genomic distributions that correlate with chromosomal organization.

Most species we analyzed, like those in subgroups 1, 2, 4, and 5, have relatively small amounts of satellite DNA in their genome (< 1.5% on average). Moreover, in most of these species, satellite DNA is dispersed throughout the euchromatic arms, with few repeats being specifically concentrated at pericentromeric/centromeric regions. Even in those species with higher amounts of satellite DNA (> 5%), such as *D. sechellia* (6.4%; subgroup 1), *D. jambulina* (5.8%; subgroup 2), and the species in subgroup 6 (5.4–10%), such repeats are mostly found spread across the euchromatic arms. Generally, simple satellite sequences, defined here as having a monomeric length smaller than 50 bp, constitute significant shares (median: 20%) but are less prevalent in the assemblies compared to complex (≥ 50 bp) satellites (Additional file 1: Fig. S5A).

In contrast with the general trend for the *Drosophila* genus, the genomes of species in subgroup 3 (except for *D. ananassae*) contain high satellite DNA content (~ 7% on average) (Fig. 1G), which is also highly enriched (median: 98%) for complex repeats (Additional file 1: Fig. S5A). In addition to being found dispersed across the euchromatic arms, the satellite DNA forms well-defined, large, and highly continuous (up to 2 Mb) domains that are characterized by high satellite DNA content and are embedded in the core of the TE-rich pericentromeric regions of each chromosome. This is the case for the metacentric chromosomes formed by the fused B/C and D/E Muller elements, as well as for the newly formed metacentric domains at elements A and F (X and 4th chromosome, respectively). Remarkably, and in contrast to the other *Drosophila* species analyzed to date, in which centromeres are mostly defined by transposon arrays [21, 58], the structure of the chromosomes in the species of subgroup 3 is reminiscent of that characterized in the model plant *Arabidopsis thaliana* and in mammalian genomes [59–62]. In these species, large arrays of satellite DNA are found at the centromeric domains of chromosomes, which are occupied by CENH3/CENP-A and assemble the kinetochore [59–64].

Since satellite DNA is particularly difficult to assemble, we set out to test whether the new scaffolded genome assemblies were of sufficient quality to identify known satellite repeats. The best-studies species in this regard is *D. melanogaster*, so we attempted to identify the presence of several well-characterized sequences, namely complex repeats 1.688, Rsp and rDNA intergenic sequence (IGS), as well as the simple satellites dodeca and prodsat [22]. For this analysis, we compared the results obtained in the new *D. melanogaster* assembly to that of the reference genome *dm6* (Additional file 1: Fig. S5B,C). While we observed a high degree of similarity in localization of most known satellite sequence between the two assemblies, the results revealed that the new assembly contained more repeats than the reference genome. Notably, the new assembly contains arrays of the minisatellites dodeca and prodsat at the pericentromeric regions of the Muller elements D/E (i.e., chromosome 3), which in turn are not present in the *dm6* assembly. Importantly, previous work focusing on centromeric sequences revealed thar dodeca and prodsat satellites are located at or directly adjacent to the functional centromere of chromosome 3 [21]. Furthermore, in comparison to the reference genome, the A element (i.e., X chromosome) of the new assembly contains an extended repeat-rich region that encompasses additional rDNA IGS sequences, which are known to be located at the heterochromatin of the X chromosome (Additional file 1: Fig. S5B,C). Finally, the new scaffolded genome also showed an improved assembly of telomeres, as indicated by the mapping of HTT (Het-A, TAHRE, TART) transposable elements to the chromosome ends [15]—in contrast to the reference genome. Together, these results confirm that the combination of ONT sequencing and HiC scaffolding produces high-quality assemblies that show sufficient completeness to study the distribution of satellite DNA.

### Satellite DNA structure in the ananassae subgroup

**Fig. 6:**
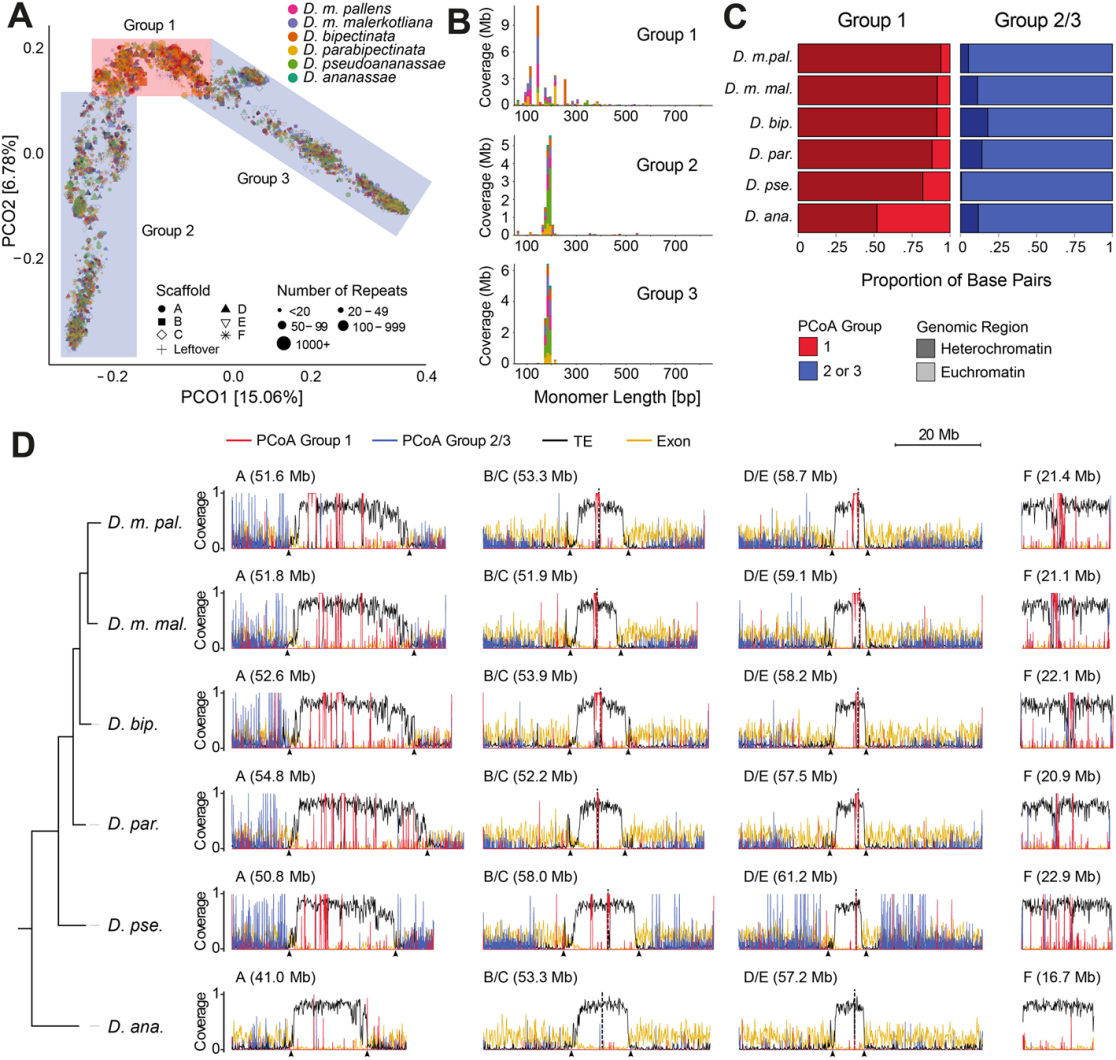
Satellite DNA analysis in *ananassae *subgroup.** A** Principal coordinate analysis (PCoA) plot on multiple sequence alignments for satellite array consensus sequences. Each point represents an array with its shape according to the scaffold/Muller element it is located on, colored by species, and its size representing the number of repeats in the array. Colored squares define three groups of Satellite DNA: red square for group 1; blue squares for groups 2 and 3. **B** Length distribution of satellite DNA repeat monomers in PCoA groups 1–3. Bar colors represent species as depicted in **A**. **C** Sequence (bp) proportion of satellite DNA from PCoA group 1 and combined PCoA groups 2 and 3 in the heterochromatic (dark) and euchromatic (light) compartments as defined by TE density (see Methods). **D** Distribution of PCoA group 1 satellite DNA (red), combined PCoA groups 2 and 3 satellite DNA (blue), TEs (black), and gene exons (yellow) across the chromosomes of the species in the *ananassae* subgroup. Bins of 100 kb were used for TEs and exons, while 30-kb bins were used for satellite DNA

Given the abundance and diversity of satellite DNAs identified by TRASH in the genomes of the *ananassae* subgroup, we first examined how the different arrays (tandem repeat blocks of satellite monomers) relate to one another in terms of divergence of their monomeric consensus sequence. To reduce the high dimensionality of the data, we performed principal coordinates analysis (PCoA) on a matrix of similarity scores obtained from multiple alignments (Fig. 6A). To preserve the wealth of information provided by TRASH, each data point (i.e., an individual array) was traced back to the species they originated from, the Muller element they were located at, and the size of the original array (in terms of number of monomer repeats). First, this analysis revealed that, at the consensus sequence level, most of the satellite DNA array content is shared between all the species in this subgroup, including the satellite DNA-poor *D. ananassae.* This was highlighted by the highly overlapping PCoA distributions within the subgroup (Fig. 6A)*,* which indicates that, globally, most of the diversity of satellite DNA sequences in this subgroup has been conserved on an evolutionary timescale of about 8.1 to 10.9 MY. Second, the PCoA distribution revealed three potential clusters of satellite DNA based on similarity scores. Closer inspection of the repeat length distribution revealed that satellite repeats from clusters 2 and 3 were highly similar in monomer length, with a narrow and discrete peak at 170–190 bp (Fig. 6B). Based on this similar monomer length profile and the fact that we did not apply any treatment to the consensus sequences before performing multiple alignments, we hypothesized that the subdivision between clusters 2 and 3 may be a by-product of the strandedness of consensus sequences. In line with this, manual inspection revealed that reverse complemented sequences from cluster 2 were consistently present in cluster 3, and vice versa. On the other hand, the arrays on cluster 1 showed distinct sequence consensus diversity and a broader monomer length distribution (centered on a prominent 140 bp peak) compared to clusters 2 and 3, forming a bona fide distinct group.

In light of the sequence and monomer length dichotomy revealed by the PCoA analysis, we determined the chromosomal distribution of the satellite DNA arrays on clusters 1 and 2/3 in each species of the *ananassae* subgroup (Fig. 6C,D). Strikingly, we observed that satellite DNA arrays from cluster 1 were highly enriched in pericentromeric/centromeric regions, while arrays from clusters 2/3 were almost exclusively distributed across the euchromatic arms (Fig. 6C). The only exception to this stereotyped distribution is provided by the satellite DNA-poor *D. ananassae*, for which cluster 1 arrays were not disproportionally found at heterochromatic regions. Yet, for all the other species in the *ananassae* subgroup, euchromatic satellite arrays were shorter in length, distributed throughout the chromosome arms and away from euchromatic-pericentromeric borders, and found within intergenic regions (Fig. 6D). Conversely, pericentromeric satellite arrays formed longer and highly contiguous blocks that are located in the middle of the pericentromeric regions, surrounded by TEs, and are located away from the euchromatin-heterochromatin border. This distinct division of pericentromeric and euchromatic satellites, and the distribution of genes and TEs, creates highly structured chromosome landscapes in all species in this subgroup, except for *D. ananassae* (Fig. 6D). Notably, a similar structure is observed on the more TE-rich and gene-poor F-element, with a highly continuous centromeric satellite domain closer to the middle of the chromosome.

**Fig. 7:**
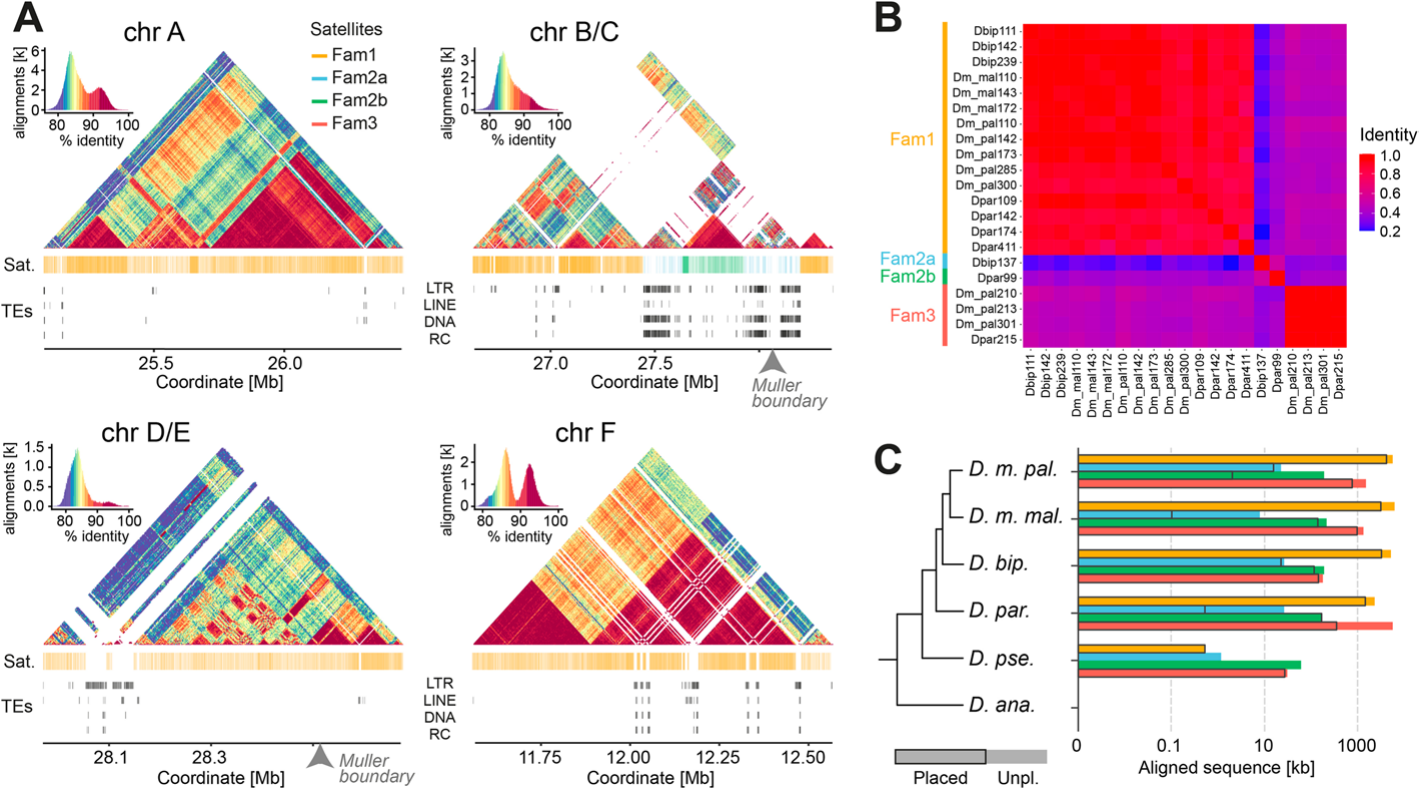
Analysis of the heterochromatic satellite DNA arrays in the *ananassae *subgroup.** A** Higher-order structure analysis of long satellite DNA arrays in *D. bipectinata*. StainedGlass sequence identity heatmap of putative centromeric regions of chromosomes A, B/C, D/E, and F, using a window size of 1 kb. Histograms at the top left show the assignment of colors to sequence identity values for each heatmap. Blast alignment hits of satellite DNA families and TE annotations are shown below. **B** Identity heatmap of multiple sequence alignments of consensus sequences of peri/centromeric satellite DNA arrays. **C** Combined lengths (kb) of all sequence alignments of satellite DNA families 1, 2a/b, and 3 in the genomes of the *ananassae* subgroup. Dispersed, very short alignments (< 50 bp) were filtered out

While the arrangement of pericentric/centromeric satellite arrays in the species of subgroup 3 is unusual in the *Drosophila* genus, it is reminiscent of the distribution in *Arabidopsis* and mammals [59–62]. Indeed, the human and the *Arabidopsis* centromeres are characterized by large arrays of satellite repeats that are organized in a highly ordered structure [20, 61]. To determine whether the peri/centromeric satellite arrays in the species of subgroup 3 are organized in highly ordered repeat units, we used StainedGlass to visualize the distribution of pairwise similarity inside the peri/centromeric arrays (Fig. 7A) [65]. This analysis revealed that, with the exceptions of *D. ananassae* and *D. pseudoananassae*, the peri/centromeric satellite DNA domains in each chromosome in each subgroup 3 species are defined by a highly ordered structure of highly identical repeats (Figs. 7A, Additional file 1: Fig. S6-8), similar to what was described in *Arabidopsis* and humans. As for *Arabidopsis*, these arrays are sporadically interrupted by transposable element insertions of various types (LTR retrotransposons, DNA transposons, LINEs, and rolling circle TEs).

To further investigate the relationship between the different satellite arrays populating the peri/centromeric regions in the genomes of the *ananassae* subgroup, we performed multiple alignment comparisons and phylogenetic analysis with the consensus sequences of peri/centromeric satellite DNA arrays, as defined by TRASH. The results revealed that most of the peri/centromeric arrays in the putative centromere regions, which all consist of complex sequences, are provided by a single family of satellite repeats (referred to as “Fam1”) that encompasses multiple variants in each species. The variants range from 109 to 411 bp in monomer length and are likely derived from a common ancestor sequence (Fig. 7B). This family is the most prevalent in abundance and distribution and can be found in the assemblies (and raw genome sequencing data) of all species that emerged after the speciation of *D. ananassae* (Figs. 7C, Additional file 1: Fig. S5D, Additional file 4: Table S3). With the notable exception of chromosome F in *D. parabipectinata* (Additional file 1: Fig. S8), this family is present in the peri/centromeric regions of all chromosomes of subgroup 3 species after the split from *D. pseudoananassae* (Figs. 7A, Additional file 1: Fig. S6-8).

In addition to the global prevalence of “Fam1,” two other satellite families occupy a significant share of the peri/centromeric domains of subgroup 3 species and are worth noticing. The first is “Fam2,” which we further distinguished in “Fam2a” (137 bp monomers) and “Fam2b” (99 bp monomers) based on sequence similarity (Fig. 7B). In peri/centromeric regions, “Fam2a” is only found on chromosome B/C in *D. bipectinata*, while arrays of “Fam2b” can be detected in *D. bipectinata*, *D. parabipectinata*, and *D. m. malerkotliana* putative centromeres (Figs. 7A, Additional file 1: Fig. S6-8). Importantly, “Fam2a” and “Fam2b” repeats are detected in the genomes of all species of the sister lineage of *D. ananassae* (Figs. 7C, Additional file 1: Fig. S5D, Additional file 4: Table S3)*.* These results suggest that, similar to the “Fam1” satellites, the common ancestor of the “Fam2” satellites emerged after the speciation of *D. ananassae*, but before the radiation of the remaining species.

The last notable family, “Fam3,” with monomer lengths between 210 and 301 bp, is only found in the peri/centromeric domains of the F chromosome of *D. parabipectinata* and *D. m. pallens* among putative centromeres (Additional file 1: Fig. S6,8). In *D. m. pallens*, the highly structured domain in the F chromosome is partitioned between the satellite repeats of this family and a satellite array from a variant of “Fam1.” In *D. parabipectinata*, however, the entire structured domain is provided by “Fam3” repeats. These results suggest that, while this satellite family was already present in the F chromosome of the common ancestor of *D. parabipectinata* and *D. m. pallens*, it was likely lost at least twice in evolution (in *D. bipectinata* and *D. m. malerkotliana*). In fact, similar to the other satellite families, “Fam3” repeats are found in all genomes of the *ananassae* subgroup besides that of *D. ananassae* (Figs. 7C, Additional file 1: Fig. S5D, Additional file 4: Table S3). Interestingly, “Fam1” and “Fam2b” sequences are completely absent from euchromatin in all species, and “Fam2a” sequences are only found in small and partial traces in euchromatic domains. On the other hand, “Fam3” sequences are consistently present in euchromatin, albeit in much smaller amounts compared to heterochromatin (Additional file 4: Table S3). Intriguingly, among the highly abundant families (“Fam1” and “Fam3”), “Fam3” is distinctly enriched at the metacentric A and F elements in most species, while “Fam1” is more equally distributed among Muller elements (Additional file 4: Table S3).

Altogether, our results suggest that the emergence and radiation of novel satellite DNA sequences in the *ananassae* subgroup occurred after the speciation of *D. ananassae.* Indeed, no remnants of these satellite DNA sequences could be identified in the *D. ananassae* genome, or in the raw genome sequencing data (Figs. 7C, Additional file 1: Fig. S5D, Additional file 4: Table S3), which is poor in tandem repeats in general (Figs. 1G, 5) and not enriched for complex satellites (67%) relative to the other species (Additional file 1: Fig. S5A). Moreover, while a small amount of “Fam1-3” satellite DNA could be identified in *D. p. pseudoananassae* (0.093 Mb), their genomic abundance is orders of magnitude lower compared to the remaining species of its sister lineage (5.6–8.2 Mb; Fig. 7C, Additional file 4: Table S3). These results suggest a dynamic, stepwise process by which the novel satellite DNA sequences of “Fam1-3” emerged after the split from *D. ananassae*, but only after the speciation of *D. p. pseudoananassae* did these satellite sequences expand to form highly contiguous and structured centromeric satellite domains. In an alternative hypothesis, *D. p. pseudoananassae* could also have lost the vast majority, i.e., ~ 99% compared to its sister species, of satellite sequences that were previously gained in this lineage.

## Discussion

Combining the strengths of long-read sequencing with chromosome capture technology [30–32], we were able to generate and annotate a large number of high-quality chromosome-level genome assemblies across the *Drosophila* genus. Focusing on the concept of Muller elements [5, 10, 11], our scaffolded assemblies follow a unified nomenclature with a consistent evolutionary logic, which facilitates broad analysis of genomic rearrangements and chromosomal organization. Indeed, the power of the genomic data set provided here is further demonstrated by our analysis of chromosome structure and satellite DNA evolution, which have been historically challenging. In this context, the combination of high-quality chromosome-level genome assemblies, phylogenetic analysis, and refined satellite DNA annotation has proven instrumental in providing a new level of understanding of the evolution of the repetitive component of the genome and sheds light on the evolutionary dynamics of higher-order repeat structures. Given the current efforts in producing high-quality chromosome-level genome assemblies across the tree of life [27–29, 66], we anticipate that the application of the framework we presented here has the potential to revolutionize our understanding of repetitive sequence evolution in eukaryotic genomes.

Prior to the emergence of the new technologies that enabled much more contiguous genome assembly and scaffolding, e.g., to examine centromere sequences in unprecedented detail [21, 58], many uncertainties prevailed about satellite DNA in the *Drosophila* genus. Previous estimates of the genomic share of satellite DNA sequences in different species ranged from 0.5% in *D. erecta* and 5% in *D. simulans*, to 20% in *D. melanogaster* and 50% in *D. virilis* [22, 67–69]. While our results show that there are indeed considerable differences in satellite content, these are much lower, i.e., 1.4 to 10% in general and 0.1 to 2.4% for simple repeats, than the previous estimates [22]. This exemplifies the long-lasting difficulties in the analysis of repetitive DNA. Despite the repetitiveness and the evolutionary instability of centromeric DNA sequences, centromeres are functionally highly conserved and fundamental to chromosome biology [70]. Therefore, the ability to study satellite DNA in detail presents itself as a valuable opportunity to further our understanding of genome function and evolution.

Overall, the genome of *D. melanogaster* and those of other closely related species contain less satellite DNA when compared to the species in the *ananassae* subgroup, with the exception of *D. ananassae* (Fig. 1). Moreover, the satellite DNA of this subset of species in the *ananassae* subgroup is highly enriched for complex satellites (Additional file 1: Fig. S5A). In *D*. *melanogaster*, the core of centromeres was previously shown to be comprised of islands of retroelements that are flanked by arrays of short simple repeats, both of which markedly differ in sequence and composition between chromosomes [21]. Comparative analysis revealed that one of the few consistent features of *D. melanogaster* centromeres is a strong enrichment of the non-LTR retrotransposon G2/Jockey-3 family, a feature that is conserved in *D. simulans* but not in other closely related species [21, 23, 58]. In contrast, we observed that most species of the *ananassae* subgroup possess long stretches (up to 2 Mb) of complex satellite DNA arrays, forming higher-order repeat structures (HORs), with monomer lengths varying between 99 and 411 bp. Similar to what is observed in species like *Arabidopsis* [61, 62], these long stretches of satellite repeats are rarely interrupted by interspersed TEs or TE fragments. While it is possible in principle that the functional centromeres are defined by transposable elements or other repeats not contained in the new *ananassae* subgroup genome assemblies, the presence of these vast stretches of complex satellite DNA, organized into HORs, appears to be unique within the *Drosophila* genus and, if not directly, they are likely to be indirectly involved in organizing the structure of the peri/centromeric regions.

Interestingly, the three main satellite families forming higher-order repeat structures in the *ananassae* subgroup have emerged fairly recently, after the speciation of *D. ananassae* but before the separation of the other species in this clade. One might speculate that the remodelling of the two acrocentric chromosomes (Muller elements A and F) into a metacentric organization required changes in centromeric structure, and that this may have been resolved by the emergence and expansion of novel satellite DNA arrays. However, the lack of these specific satellite families, as well as an overall depletion of tandem repeats in the genome of *D. ananassae,* does not support the interdependence between the two processes. Ultimately, it remains to be determined what spurred the expansion of satellite DNA in this lineage.

Despite the central role of TEs in the centromeres of *D. melanogaster—*making up about 70% of the functional centromeric DNA [21]—our analyses suggest that this organization may not be generalisable for the entire *Drosophila* genus. In fact, closer inspection of centromeres in a very closely related sister group to *D. melanogaster *(comprising *D. simulans*, *D. sechellia*, and *D. mauritiana*) already showed a dramatic reorganization of centromeric TEs and satellite DNAs on a short evolutionary timescale [58]. In this context, the *ananassae* subgroup provides an alternative model for studying the evolution of peri/centromeric satellite DNA within the genus. While the evolution, distribution, and organization of satellite DNA in this subgroup appears unique among the species in the phylogeny studied here, it shows remarkable similarities to what is observed in the human and *Arabidopsis* genomes. The variability in the exact composition of satellite DNA families of peri/centromeric domains between species in this subgroup supports previous observations that no specific sequence features are determinant for centromere function and that presumably any heterochromatic satellite DNA arrays might be capable of acquiring centromere activity [71, 72]. In the same way, newly emerged tandem-repeat arrays in the common ancestor of *D. parabipectinata*, *D. bipectinata*, *D. m. malerkotliana*, and *D. m. pallens* might have taken over centromere function, while the same satellite DNA sequence families in *D. p. pseudoananassae* failed to do so, or alternatively might have lost their function. Crucially, the active centromere itself is ultimately determined epigenetically through CENP-A and cannot be definitively predicted on the DNA sequence level alone [73, 74]. While many aspects of this process remain to be determined, we propose that the *ananassae* subgroup provides a novel model system for satellite DNA and centromere evolution.

## Conclusions

Recent advances in long-read sequencing and chromosome capture methods enabled the reconstruction of much more complete and contiguous genome assemblies. We used this powerful combination to generate 30 chromosome-level genome assemblies across the *Drosophila* genus, for which we have further annotated genes, TEs and tandem repeats through various specialized state-of-the-art computational tools. This data set is a valuable resource for addressing key outstanding research questions in genome biology. Our comparative analyses of genome organization and genomic rearrangements demonstrate the power of this large and high-quality data set in uncovering the dynamics of chromosome evolution. Furthermore, and thanks to its quality, we were able to perform a deep investigation into peri/centromeric domains and dynamics of the repetitive component of the genome.

After long-lasting uncertainty about the nature of centromeres and genomic contribution of tandem repeats in flies [22], the first detailed analyses of *Drosophila* satellite DNA and centromeres were conducted in *D. melanogaster* and the closely related species of the *simulans* complex [21, 23, 58]. However, outside of *D. melanogaster* and its closest relatives, little was known about satellite DNA for the rest of the *Drosophila* genus. Our analyses in the *ananassae* subgroup provide valuable insights into an alternative mode of peri/centromeric domain organization in comparison to *D. melanogaster*, while showing similarities to the centromeric organization observed in *Arabidopsis* and humans [59–62]. In summary, our work demonstrates the evolutionary plasticity of satellite DNA and peri/centromeric domains, while also providing a new model for the study of centromere evolution in metazoans.

## Methods

### Fly stocks and husbandry

Stocks for *Drosophila* species were obtained from the National Drosophila Species Stock Center (NDSSC, Cornell University, USA), the KYORIN-Fly Drosophila Species Stock Center (Kyorin University, Japan), and the Bloomington Drosophila Stock Center (BDSC, Indiana University, USA). Species are listed in Additional file 2: Table S1. Fly stocks were maintained on standard cornmeal medium at 25 °C.

### HiC-seq

HiC-sequencing libraries were generated using the Arima-HiC + kit followed by the Swift Accel-NGS 2S Plus library preparation kit with some modifications. Fifty to one hundred adult female flies were collected, flash frozen, and pulverized into a fine powder using a pellet pestle. Crosslinking, lysis, chromatin fragmentation, repair and biotinylation, ligation, shearing, and pull-down were all performed according to the Arima-HiC + protocol. Libraries were generated using the Swift-Accel-NGS 2S Plus library preparation kit and PCR amplified for 8–10 cycles. The final libraries were then quality-checked and quantified for multiplexing using the Bioanalyzer High-Sensitivity DNA kit (Agilent) and Qubit dsDNA HS Assay kit (Thermo Scientific). The multiplexed libraries were sequenced as 150 bp paired-end reads on the Illumina Novaseq 6000 SP.

### RNA-sequencing

RNA-sequencing libraries were generated using the NEBNext Poly(A) mRNA Magnetic Isolation Module followed by the NEBNext Ultra II Directional RNA Library Kit for Illumina. Briefly, adult females were dissected in ice-cold PBS and 40–50 ovaries were flash-frozen and stored at − 80C until further processing. Total RNA was collected from the Lexogen TraPR Small RNA isolation columns using TRIzol LS (after small RNA isolation), and subsequently purified with the addition of chloroform and isopropanol. After purification, 2.5 µg of RNA was measured by Qubit RNA HS Assay kit (Thermo Scientific) and used for poly(A) isolation and library preparation. Libraries were amplified for 8 cycles, and quality checked and quantified for multiplexing using the Bioanalyzer High-Sensitivity DNA kit (Agilent) and Qubit dsDNA HS Assay kit (Thermo Scientific). The multiplexed libraries were sequenced as 150-bp paired-end reads on the Illumina Novaseq 6000 SP.

### HiC genome scaffolding

Paired-end HiC reads were trimmed with Trim Galore, which utilizes the trimmer tool cutadapt [75] at its core. Each read pair file was then separately mapped to the corresponding genome sequence, which was obtained for all species from Kim et al. [28] (NCBI BioProject PRJNA675888; Additional file 2: Table S1), with bwa mem [76] and a map quality (mapq) cutoff of 10. Mapped reads were then filtered and combined using Arima HiC pipeline scripts (filter_five_end.pl, two_read_bam_combiner.pl; github.com/ArimaGenomics/mapping_pipeline). After deduplication with sambamba [77], the merged bam file was converted into bed format with bedtools [78]. The final bed file was used as input for YaHS [39] for automated genome scaffolding. Contigs are separated by 100 N gaps within scaffolds. Juicer [79] was then used to produce assembly contact maps for manual curation, which was enabled through the HiC visualization tool juicebox [80]. Importantly, for most metacentric chromosomes made up by two Muller elements, we were forced to manually define the end of one chromosome arm and the beginning of the other arm to strictly adhere to the Muller element nomenclature. In such cases, the breakpoint between two Muller elements was assigned using the Juicebox tool based on the chromatin conformation data as reference, as previously described [81]. Finally, given that in a few instances different stocks were used to generate the Nanopore [28] and the HiC data (this study; see Additional file 2: Table S1), we consistently prioritized the contiguity of the originally assembled contigs (i.e., Nanopore-based) whenever we observed conflicts between the two types of data.

### Muller element allocation

Muller elements were identified by whole genome alignment with blastn [82] to the *D. melanogaster* reference genome dm6. Scaffolds were allocated to a Muller element by best overall mutual alignment, where dm6 chromosomes X, 2L, 2R, 3L, 3R, and 4 represent Muller elements A through F, respectively. The final orientation of each Muller element scaffold was determined by the concentration of TE sequences in either half as to correspond to Muller element orientations in dm6. Muller elements A, B, and D end with high TE accumulation, while C and E start with high TE abundance. For this purpose, TEs were annotated using RepeatMasker with the standard drosophila_flies_genus consensus library.

### Gene annotation

First, Trinity [83] was used for the de novo transcriptome assembly of paired-end RNA-Seq data. The resulting transcriptome was then used as input for the de novo gene annotation tool Maker [40], alongside of protein sequences from representative species *D. melanogaster*, *D. ananassae*, *D. persimilis*, *D. virilis*, and *D. willistoni*, obtained from NCBI. Within the Maker pipeline, Exonerate [84] searched for protein sequence homologies, while Augustus [85] performed ab initio gene prediction. Finally, gene orthologs across species were identified with OrthoFinder [86] and identified orthologous genes were named corresponding to *D. melanogaster* homologies.

### Transposable element annotation

HiTE [41], which performs dynamic boundary adjustment for full-length TE detection, was used for de novo transposon annotation with options ‘–annotate 1 –plant 0’. RepeatModeler [87], as part of the HiTE pipeline, generated the resulting reference TE libraries, which were then used by RepeatMasker for final annotation. RepeatMasker output files were then converted to bed files, which in turn were binned into 100-kb bins for chromosomal TE density plotting. These were also used to predict pericentromeric heterochromatin regions for each Muller element, seeking continuous blocks of 100-kb bins with at least 20% TE share, while allowing small dips below 20%, but ignoring small TE-rich islands within euchromatin (such as TE/piRNA clusters 38C, 42AB, and others in *D. melanogaster*).

#### HiC-based Muller element organization and compartment analysis

First, HiC sequencing data were re-mapped against the final scaffolded genome assemblies as described above. Contacts between Muller elements were then counted in the final bed file of combined paired-end reads and normalized through division by the square root of the product of Muller element lengths. Additionally, physical contacts between Muller elements were visually confirmed with HiC contact maps using juicebox [80]. PCA eigenvectors for the identification of A and B compartments were calculated on Pearson correlation matrices of HiC maps after *z*-score normalization, using the python library scikit-learn, for whole genomes as well as for individual Muller elements.

### Genome rearrangement analysis

For the identification of syntenic blocks between species, we used the R package GENESPACE [49], which relies on OrthoFinder [86] for ortholog identification, DIAMOND [88] for protein sequence alignment and MCScanX [89] for gene synteny detection. The resulting syntenic block coordinates for all species comparisons were then extracted and compared to evolutionary/genetic distance values provided through the phylogenetic species tree, which was generated by OrthoFinder for all 30 species. Finally, the syntenic breakpoints, i.e., syntenic block borders, were identified in *D. melanogaster* and compared to the remaining 29 species to plot breaks per gene bins.

### Tandem repeat annotation and analysis

Tandem repeats were identified for genomic assemblies of 30 *Drosophila* species using TRASH [42] with default settings. As validation, a list of representative satellite repeats in *D. melanogaster* obtained from the literature [90] was individually cross-checked against the repeats identified by TRASH. For satellites ≤ 12 bp, exact matches were sought. For longer satellites, MegaBLAST [91] with default parameters was used to identify TRASH output sequences with high similarity. Search queries were retrieved from GenBank: X78946 (IGS), AJ275930.1 (1.688 family), and U53806.1 (Rsp). Requiring ≥ 80% query coverage for a successful match, all representative satellites in *D. melanogaster* were identified de novo by TRASH.

Principal coordinate analysis (PCoA) was performed on multiple sequence alignments generated for all array consensus sequences with length ≥ 50 bp without prior processing or filtering, e.g., removal of potential reverse complements. Sequence alignments were generated using MAFFT [92] with default settings. Distance matrices were computed for each using the dist.alignment function from the “seqinr” R package [93]. An identity matrix was used calculate pairwise distances, with alignment gaps counted in the identity measure. PCoA was performed using the cmdscale R function. Sequence identity maps for representative heterochromatic regions were obtained using StainedGlass [65] with default settings.

For each species, heterochromatic arrays were sorted by consensus length. Arrays with consensus lengths within 1 bp were grouped. Groups with > 5 kb total coverage in the identified regions were each aligned using MUSCLE in MEGA11 [94] with default settings. Alignments were corrected manually as required. This included considering the reverse complement for proper alignment. Where necessary, unrelated arrays were identified by visual inspection and aligned separately. For each alignment, an overall consensus was derived by taking the highest-frequency nucleotide at each position. Pairwise identities were calculated on multiple sequence alignments of consensuses using the “seqinr” R package [93], excluding alignment gaps.

## Supplementary Information

Additional file 1. Supplementary figures Fig. S1 to Fig. S8.

Additional file 2. Table S1: Summary of samples and data used and generated in this study.

Additional file 3. Table S2: Continuous regions with low number of synteny breakpoints within bins of 10 genes across the D. melanogaster genome when compared to the remaining 29 species.

Additional file 4. Table S3: Combined lengthsof all sequence alignments of satellite DNA families 1, 2a/b and 3 in the genomes of the ananassae subgroup in euchromatin/heterochromatin and per chromosome.

## Data Availability

Hi-C and RNA-seq data generated during this study is deposited at the NCBI sequence read archive (SRA) under BioProject number PRJNA1119277 [95]. Genome assemblies generated in this study are deposited at NCBI Datasets (as 'Third Party Data Assembly'), and can be found under the "WGS master" heading within the same BioProject number. Publicly available Nanopore data for all 30 species are found under BioProject number PRJNA675888 [96]. Publicly available HiC data for 8 Drosophila species (Additional file 2: Table S1) [97–104] supplemented our datasets. Custom code used in this study, as well as all genome annotations (gene, transposable elements, and tandem repeats), are deposited at Github (https://github.com/d-gebert/30_fly_genomes) [105] and Zenodo (10.5281/zenodo.14737546) [106] and are licensed under the MIT License. Any additional information required to reanalyse the data reported in this paper is available from the lead contact upon request. Further information and requests for resources or reagents should be directed to the lead contact Felipe Karam Teixeira (fk319@cam.ac.uk).
