# Supplementary material for: Analysis of 30 chromosome-level Drosophila genome assemblies reveals dynamic evolution of centromeric satellite repeats
Source: Genome Biol. 2025 Mar 18;26:63. doi: 10.1186/s13059-025-03527-4 (PMC11917152; doi:10.1186/s13059-025-03527-4)
Supplement: Supplementary file 1 — Additional file 1. Supplementary figures Fig. S1 to Fig. S8. [file 13059_2025_3527_MOESM1_ESM.pdf]

Supplementary Figures

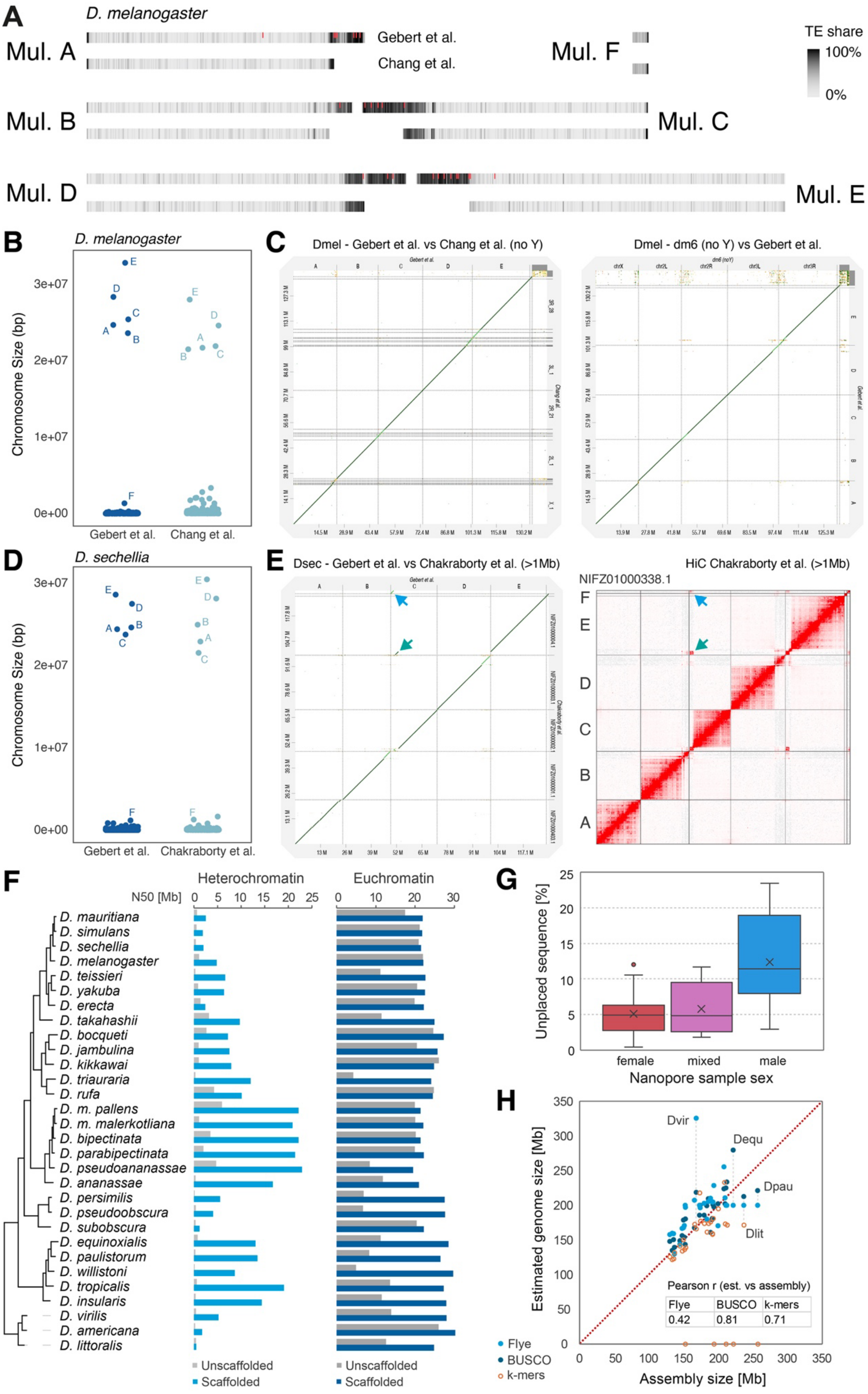

Figure S1: Quality assessment of HiC-scaffolded genome assemblies and comparison to previously published assemblies. (A) Comparison of HiC-scaffolded *D. melanogaster* genome assembly generated in this study, to PacBio-generated assembly published by Chang et al. 2019 [21]. Muller elements of both assemblies, with TE-density shown in 100 kb bins (light gray to black: low to high), are shown side by side with aligned euchromatic arms. Gaps are indicated by red bars. (B) Distribution of sequence sizes in HiC-scaffolded *D. melanogaster* genome assembly and Chang et al. 2019 assembly [21]. (C) Left: sorted alignment plot of HiC-scaffolded *D. melanogaster* assembly and Chang et al. 2019 assembly (without Y chromosomal sequences) [21]. Right: sorted alignment plot of *dm6* (without Y chromosomal sequences) and HiC-scaffolded *D. melanogaster* assembly. (D) Distribution of sequence sizes in HiC-scaffolded *D. sechellia* genome assembly and Chakraborty et al. 2021 assembly [23]. (E) Left: sorted alignment plot of HiC-scaffolded *D. sechellia* assembly and Chakraborty et al. 2021 assembly (only sequences > 1 Mb) [23]. Right: HiC contact map of Chakraborty et al. 2021 *D. sechellia* assembly [23]. Besides Muller elements A-F, the Chakraborty et al. 2021 *D. sechellia* assembly contains one additional scaffold larger than 1 Mb (NIFZ01000338.1) [23]. Arrows indicate disagreements between genome assembly and species-specific HiC-based contact map. (F) N50 values of unscaffolded assemblies published by Kim et al. 2021 [28] (gray) and assemblies scaffolded in this study (blue) in Mb (million base pairs). Left: heterochromatic regions (approximated by repeat density and distribution). Right: euchromatic regions. (G) Shares of genomic sequence that were not placed in any Muller element grouped by sex of samples used in Nanopore sequencing. (H) Correlation of estimated genome sizes and assembly sizes, as calculated in Kim et al. 2021 [28].

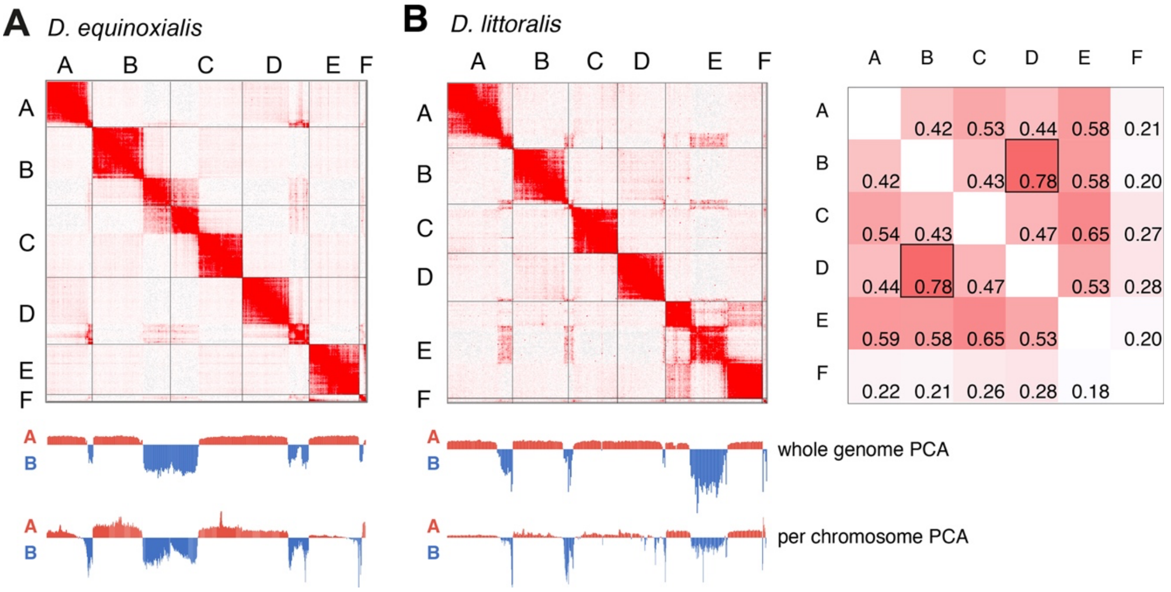

Figure S2: HiC data contact visualization and Muller element contact quantification for *D. equinoxialis and D. littoralis*. (A) HiC contact map for scaffolded genome assembly of *D. equinoxialis*. Distribution of A/B compartment along the genome is shown below the HiC maps (PCA eigenvectors for whole genome and Muller element-specific PCA). (B) Left: HiC contact map for scaffolded genome assembly of *D. littoralis*. Right: Normalized contact intensity values between Muller elements. Individual squares for each pairwise comparison are coloured on a white-to-red scale representing low-to-high contact values. Squares containing the highest values for each pair of Muller elements are highlighted by black borders.

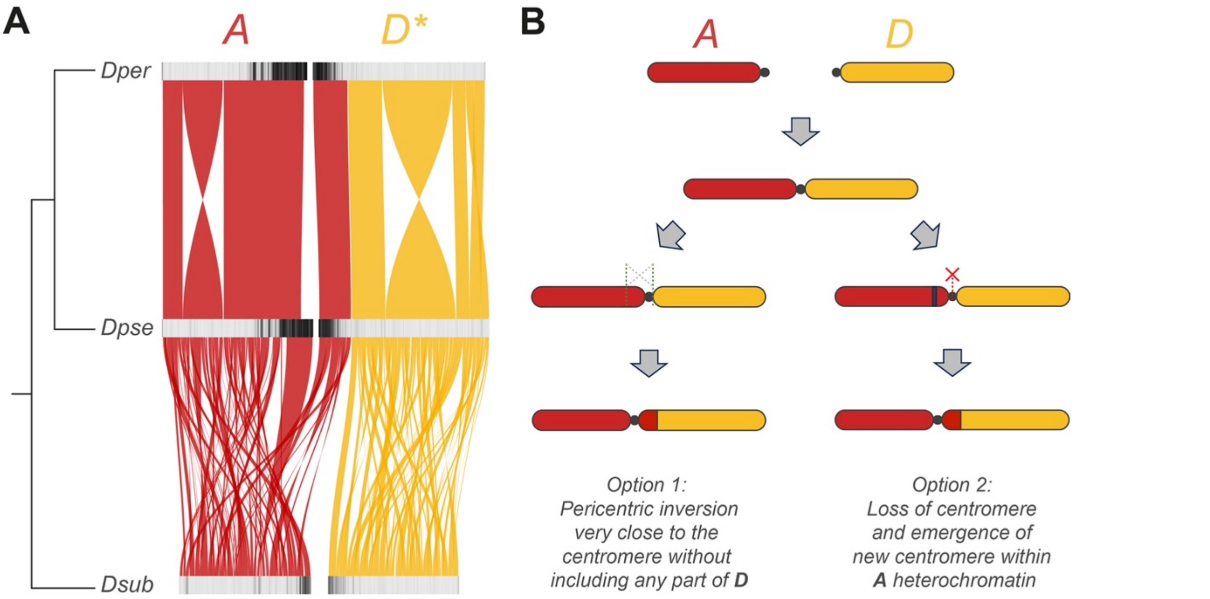

Figure S3: Synteny of A and D elements between *D. persimilis*, *D. pseudoobscura* and *D. subobscura*. (A) TE densities per 200 kb bins are shown with black (high TE density) to light gray (low TE density) scales. *: D element is shown in reverse orientation compared to convention in this study to correspond to actual orientation in linked A/D chromosomes of *D. persimilis* and *D. pseudoobscura*. (B) Hypothetical paths for the evolution of the linked A/D chromosomes of *D. persimilis* and *D. pseudoobscura* to explain the non-reciprocal transfer of genetic material from Muller A to D.

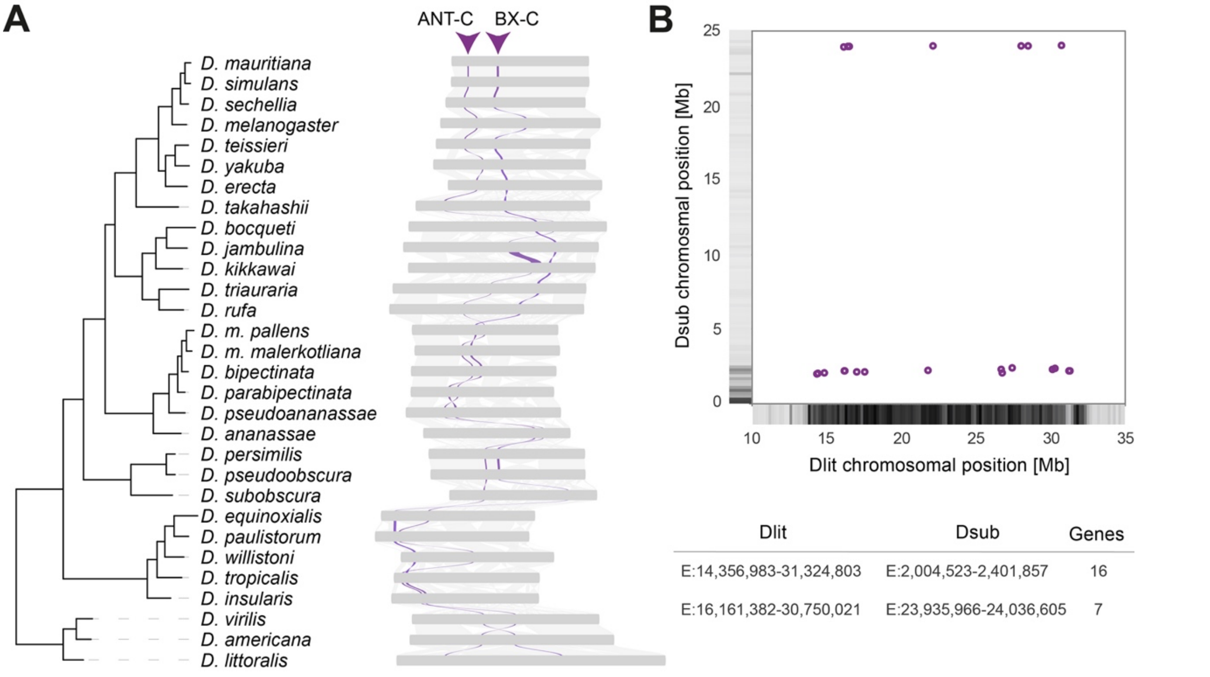

Figure S4: Tracing of chromosomal locations of *Hox* gene clusters in the genomes of *Drosophila* species and details in overlapping syntenic blocks between *D. littoralis* and *D. subobscura*. (A) Synteny of ANT-C (Antennapedia cluster) and BX-C (bithorax cluster) on Muller element E throughout phylogeny of 30 *Drosophila* species. Purple: ANT-C and BX-C. Gray: Background syntenies. (B) Top: Correlation plot between positions of orthologous genes in *D. littoralis* (Dlit) and *D. subobscura* (Dsub) within two syntenic blocks, overlapping within the heterochromatic region of the E element in *D. littoralis*. Plot axes contain TE density (light gray to black: low to high) in bins of 100 kb. Bottom: Syntenic block coordinates and number of orthologous genes in each block.

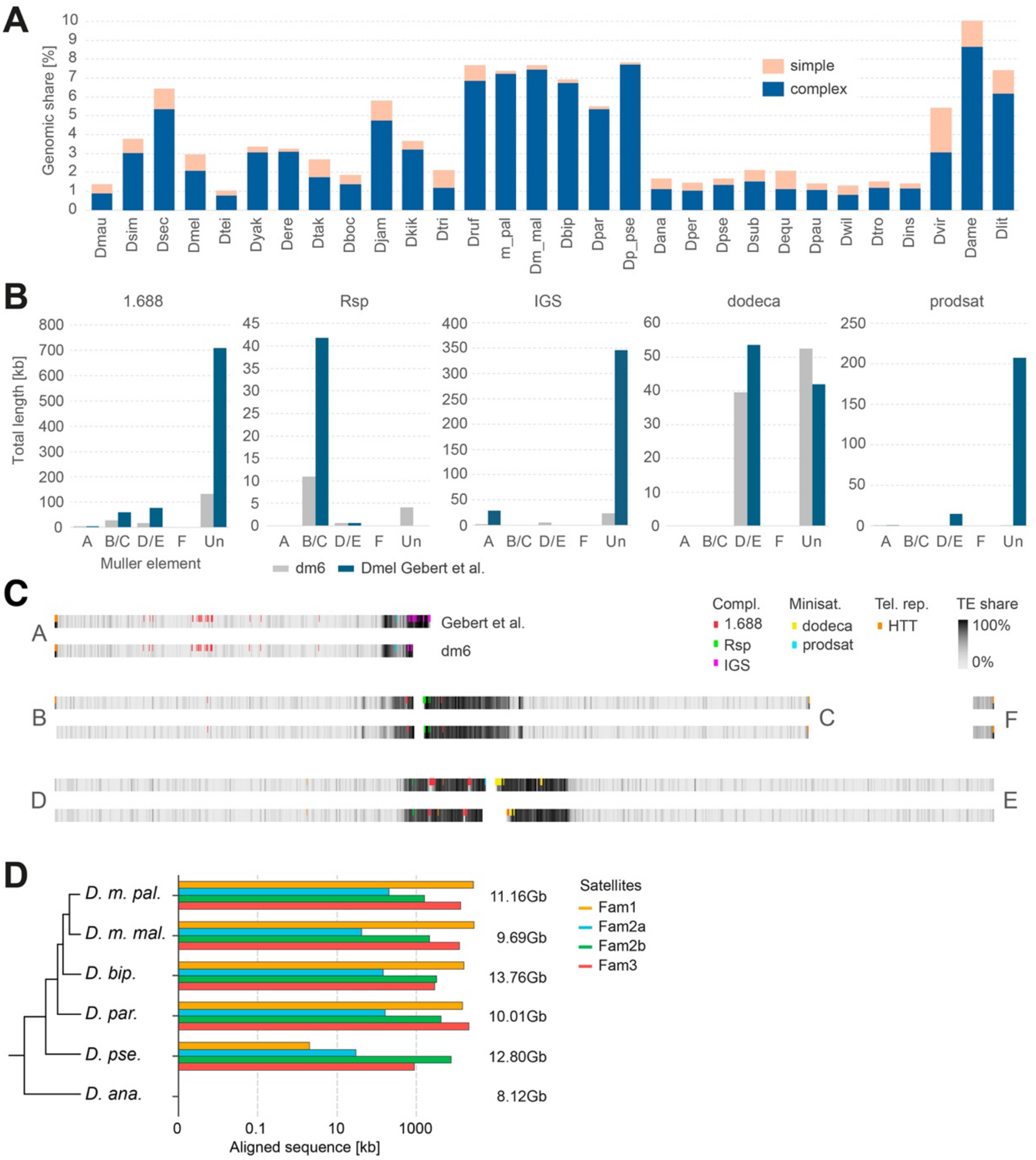

Figure S5: Presence and localisation of satellite DNAs. (A) Share of satellite DNA as percentage of genomic sequence that are classified as simple (<50 bp monomers, orange) or complex (≥50 bp monomers, blue) satellite sequence. (B) Distribution of known *D. melanogaster* satellite DNAs 1.688, Rsp, IGS (rDNA), dodeca and prodsat in the HiC-scaffolded *D. melanogaster* assembly (this study) compared to reference genome *dm6*. (C) Locations of satellite DNA sequences 1.688, Rsp, IGS (rDNA), dodeca and prodsat, as well as telomeric TEs (HeT-A, TAHRE, TART: HTT), on chromosome maps (Muller elements A-F) in the HiC-scaffolded *D. melanogaster* assembly (this study) compared to reference genome *dm6*. Density of TEs (light gray to black: low to high) are shown in bins of 100 kb. Compl.: complex repeats; Minisat.: minisatellites; Tel. rep.: telomeric repeats. (D) Combined lengths (kb) of all sequence alignments of satellite DNA families 1, 2a/b and 3 in the unassembled ONT sequencing reads of the *ananassae* subgroup. Dispersed, very short alignments (<50bp) were filtered out.

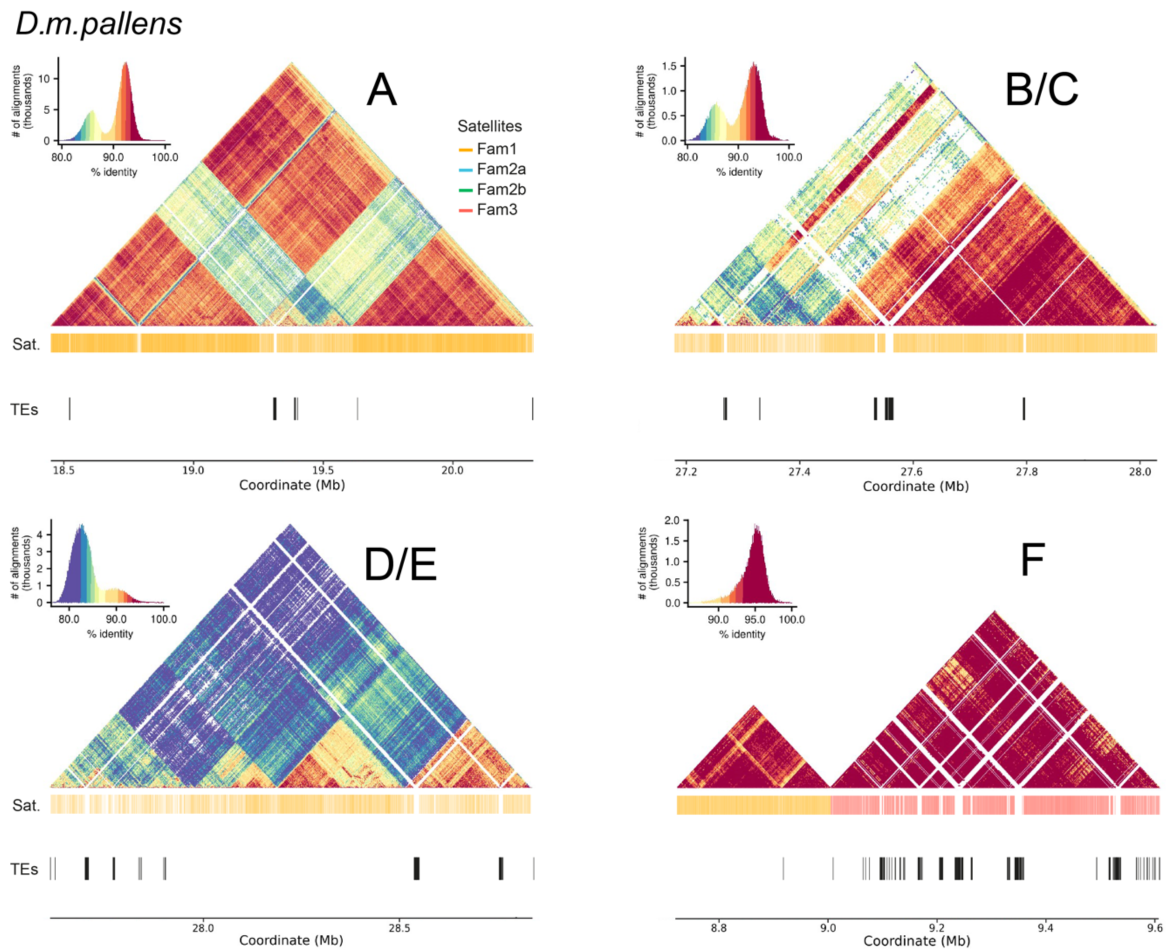

Figure S6: Higher order structure analysis of long satellite DNA arrays in *D. m. pallens*. StainedGlass sequence identity heatmap of putative centromeric regions of chromosomes A, B/C, D/E and F. Histograms at the top left show the assignment of colors to sequence identity values for each heatmap. Blast alignment hits of satellite DNA families and TE annotations are shown below.

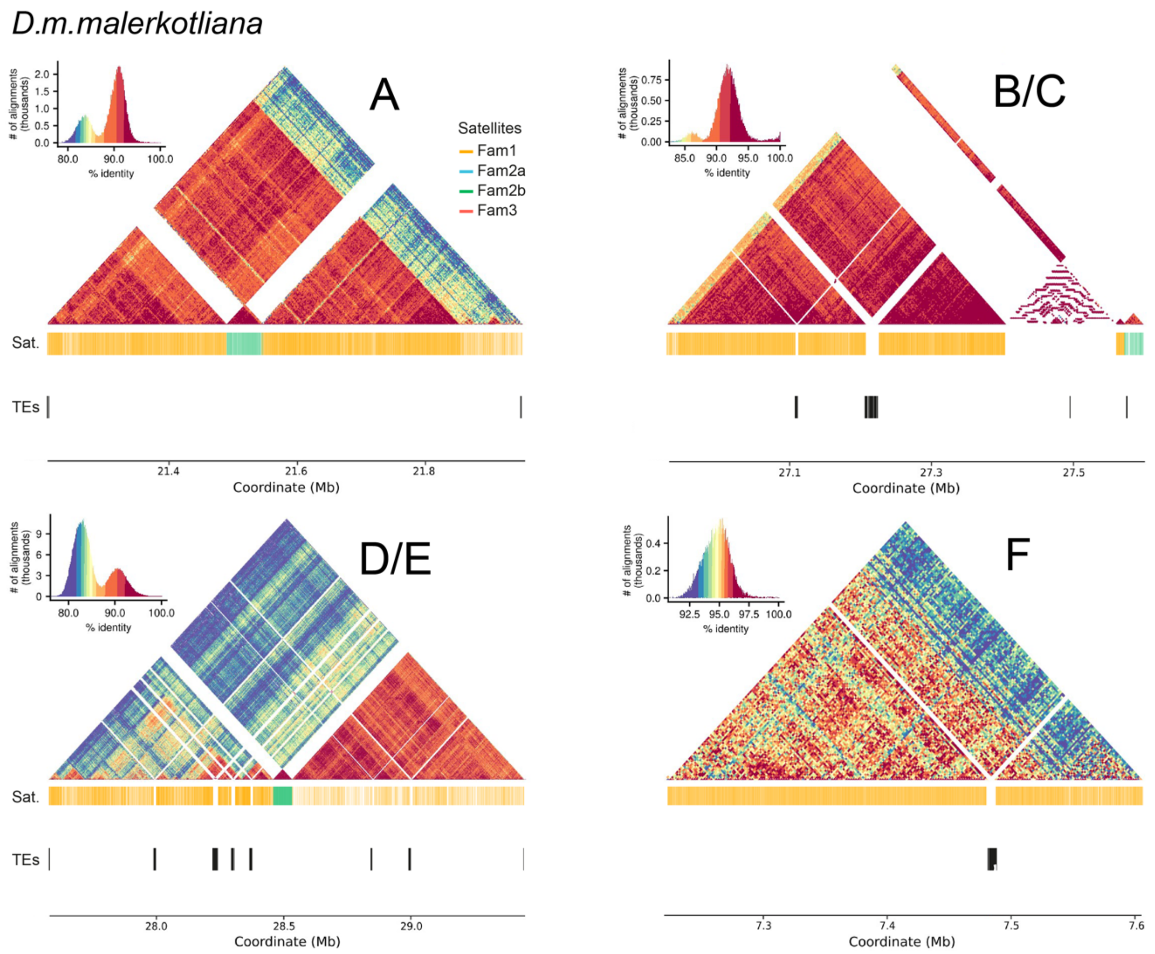

Figure S7: Higher order structure analysis of long satellite DNA arrays in *D. m. malerkotliana*. StainedGlass sequence identity heatmap of putative centromeric regions of chromosomes A, B/C, D/E and F. Histograms at the top left show the assignment of colors to sequence identity values for each heatmap. Blast alignment hits of satellite DNA families and TE annotations are shown below.

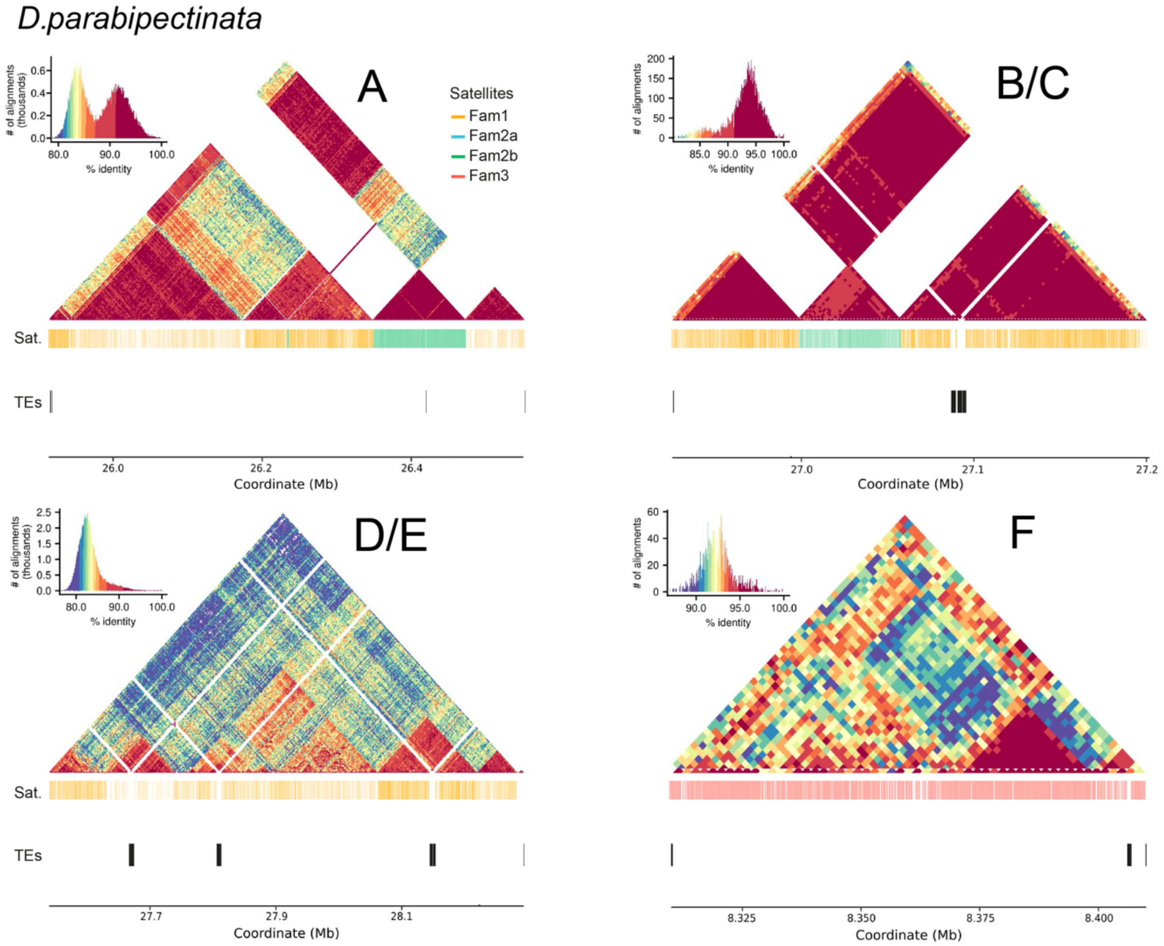

Figure S8: Higher order structure analysis of long satellite DNA arrays in *D. parabipectinata*. StainedGlass sequence identity heatmap of putative centromeric regions of chromosomes A, B/C, D/E and F. Histograms at the top left show the assignment of colors to sequence identity values for each heatmap. Blast alignment hits of satellite DNA families and TE annotations are shown below.
